# RNA-Binding Protein Rnc1 Regulates Cell Length at Division and Acute Stress Response in Fission Yeast through Negative Feedback Modulation of the Stress-Activated Mitogen-Activated Protein Kinase Pathway

**DOI:** 10.1128/mBio.02815-19

**Published:** 2020-01-07

**Authors:** Francisco Prieto-Ruiz, Jero Vicente-Soler, Alejandro Franco, Elisa Gómez-Gil, Marta Sánchez-Marinas, Beatriz Vázquez-Marín, Rosa Aligué, Marisa Madrid, Sergio Moreno, Teresa Soto, José Cansado

**Affiliations:** a Yeast Physiology Group, Departmento de Genética y Microbiología, Facultad de Biología, Universidad de Murcia, Murcia, Spain; b Department of Biomedical Sciences, Facultat de Medicina, Universidad de Barcelona, Barcelona, Spain; c Instituto de Biología Funcional y Genómica (IBFG), Consejo Superior de Investigaciones Científicas, Universidad de Salamanca, Salamanca, Spain; Universidad de Córdoba

**Keywords:** MAP kinases, RNA-binding proteins, fission yeast

## Abstract

RNA-binding proteins (RBPs) play a major role during control of mRNA localization, stability, and translation and are central to most cellular processes. In the fission yeast Schizosaccharomyces pombe, the multiple K homology (KH) domain RBP Rnc1 downregulates the activity of the cell integrity pathway (CIP) via stabilization of *pmp1*^+^ mRNA, which encodes the Pmp1 phosphatase that inactivates Pmk1, the mitogen-activated protein kinase (MAPK) component of this signaling cascade. However, Rnc1 likely regulates the half-life/stability of additional mRNAs. We show that Rnc1 downregulates the activity of Sty1, the MAPK of the stress-activated MAPK pathway (SAPK), during control of cell length at division and recovery in response to acute stress. Importantly, this control strictly depends on Rnc1’s ability to bind mRNAs encoding activators (Wak1 MAPKKK, Wis1 MAPKK) and downregulators (Atf1 transcription factor, Pyp1 and Pyp2 phosphatases) of Sty1 phosphorylation through its KH domains. Moreover, Sty1 is responsible for Rnc1 phosphorylation *in vivo* at multiple phosphosites during growth and stress, and these modifications trigger Rnc1 for proper binding and destabilization of the above mRNA targets. Phosphorylation by Sty1 prompts Rnc1-dependent mRNA destabilization to negatively control SAPK signaling, thus revealing an additional feedback mechanism that allows precise tuning of MAPK activity during unperturbed cell growth and stress.

## Abstract (precis)

Control of mRNA localization, stability, turnover, and translation by RNA-binding proteins (RBPs) influences essential processes in all eukaryotes, including signaling by mitogen-activated protein kinase (MAPK) pathways. We describe that in the fission yeast Schizosaccharomyces pombe the RBP Rnc1 negatively regulates cell length at division during unperturbed growth and recovery after acute stress by reducing the activity of the MAPK Sty1, which regulates cell growth and differentiation during environmental cues. This mechanism relies on Rnc1 binding to specific mRNAs encoding both enhancers and negative regulators of Sty1 activity. Remarkably, multiple phosphorylation of Rnc1 by Sty1 favors RBP binding and destabilization of the above mRNAs. Thus, posttranscriptional modulation of MAP kinase signaling by RNA-binding proteins emerges as a major regulatory mechanism that dictates the growth cycle and cellular adaptation in response to the changing environment in eukaryotic organisms.

## INTRODUCTION

RNA-binding proteins (RBPs) assemble into different mRNA-protein complexes and play key roles in posttranscriptional processes in eukaryotes, including splicing regulation, mRNA transport, and modulation of mRNA translation and decay (1). The K homology domain (KH) is found as multiple copies in many eukaryotic RBPs that coordinate the different steps of RNA synthesis, metabolism, and localization (2). Eukaryotic type I KH domains share a minimal βααβ structure with two additional α and β strands folded in a C-terminal orientation to this core motif (3). A conserved GXXG loop located between α1 and α2 helices is essential for RNA recognition and docking by KH domain-containing RBPs, and mutations in this motif fully impair its nucleic acid binding ability (2, 3).

Like all eukaryotes, the fission yeast Schizosaccharomyces pombe possesses a large number of putative RBPs (∼140), many of which (∼60%) are encoded by nonessential genes (4). Among them, Rnc1 is a KH-domain nonessential RBP that has been functionally characterized in this organism (5, 6). A main *in vivo* target for Rnc1 is *pmp1*^+^ mRNA, which encodes the dual-specificity phosphatase Pmp1, which specifically dephosphorylates and inactivates mitogen-activated protein kinase (MAPK) Pmk1, the core member of the cell integrity pathway (CIP) in fission yeast (5, 7). Rnc1 negatively regulates CIP signaling via stabilization of *pmp1*^+^ mRNA; consequently, combined mutation of the GXXG loops within each of its three KH domains results in abrogated mRNA binding and a lack of function phenotype similar to Rnc1 deletion (8). Moreover, activated Pmk1 binds and phosphorylates Rnc1 *in vivo* at a MAPK consensus phosphosite located at a threonine residue at position 50, and this posttranslational modification enhances the activity of Rnc1 to bind and stabilize Pmp1 mRNA, thus posing Rnc1 as a negative feedback loop of MAPK signaling (5, 6). However, besides *pmp1*^+^ mRNA, to date no other mRNAs have been shown to be regulated by Rnc1 *in vivo*. Intriguingly, a systematic comparative transcriptomic analysis has revealed that, with respect to wild-type cells, the number of upregulated genes in vegetatively growing *rnc1Δ* cells is much larger than those that become downregulated (77 versus 27, respectively) (4), suggesting that Rnc1 may also negatively regulate the mRNA half-life/stability of specific transcripts.

**FIG 1:**
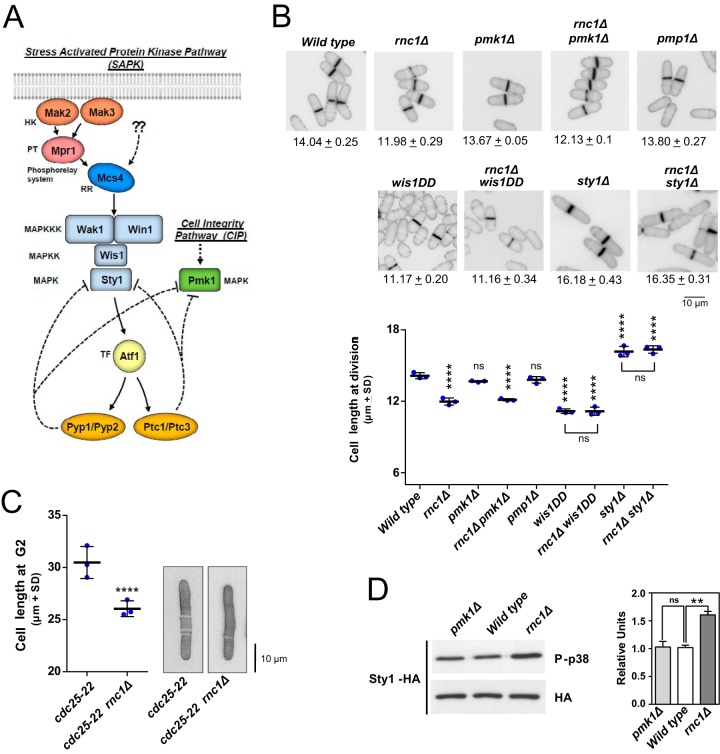
(A) The S. pombe stress-activated (SAPK) and cell integrity (CIP) MAP kinase pathways. Please see text for a detailed description of their main components and functions. (B) Cell lengths at division of S. pombe cells growing exponentially in YES medium are presented as scatter plots showing the average values ± SD (number of independent biological replicates = 3) for the wild-type and mutant strains of the indicated genotypes (number of cells ≥ 200/strain). Significant differences were assessed by Tukey’s test following one-way analysis of variance (ANOVA) for the comparisons with respective values of wild-type cells. ****, *P < *0.0001; ns, not significant. Cell morphology of each strain was analyzed by fluorescence microscopy after staining with calcofluor white. Bar, 10 μm. (C) *cdc25-22* (control) and *cdc25-22 rnc1*Δ S. pombe cultures were incubated in YES medium at the restrictive temperature (36.5°C) for 3.5 h, and cell length at G_2_ was measured and represented as scatter plots showing the average values ± SD for three independent biological replicates (number of cells ≥ 200/strain). Significant differences were assessed by Tukey’s test following one-way ANOVA for the comparisons with respective values of wild-type cells. ****, *P* < 0.0001. Cell morphology of each strain was analyzed by fluorescence microscopy after staining with calcofluor white. Bar, 10 μm. (D) S. pombe wild-type, *pmk1Δ*, and *rnc1Δ* cells expressing a genomic Sty1-HA6his fusion were grown in YES medium to mid-log phase. Activated and total Sty1 were detected with anti-phospho-p38 and anti-HA antibodies, respectively. Relative units as mean ± SD (biological triplicates) for Sty1 phosphorylation (anti-phospho-p38 blot) were determined with respect to the internal control (anti-HA blot). **, *P* < 0.005; ns, not significant, as calculated by unpaired Student’s *t* test.

The stress-activated MAPK pathway (SAPK) plays an essential role during the control of cell cycle and the general response to stress in S. pombe (Fig. 1A) (7). Once activated by dual phosphorylation at two conserved threonine and tyrosine residues by the MAPKK Wis1, Sty1, the core MAPK component of the module, moves to the nucleus and phosphorylates the bZIP domain transcription factor Atf1 to modulate the expression of the CESR (core environmental stress response) genes, which participate in the consequent adaptive cell response (Fig. 1A) (9). Besides Atf1, activated Sty1 phosphorylates multiple nuclear and/or cytoplasmic substrates, including Srk1 kinase and polo kinase Plo1, to regulate cell cycle progression at the G_2_/M transition during growth and stress (7, 10). Activated Sty1 also phosphorylates Csx1, an RBP that associates with and stabilizes *atf1*^+^ mRNA to modulate the expression of Sty1- and Atf1-dependent genes during oxidative stress, and is critical for cell survival under this specific condition (11). Importantly, both the SAPK pathway and CIP functionally cross talk, since the Sty1-tyrosine phosphatases Pyp1 and Pyp2 and serine/threonine phosphatases Ptc1 and Ptc3, whose transcriptional induction is dependent on the Sty1-Atf1 branch, also associate with and dephosphorylate activated Pmk1 *in vivo* (12). Thus, the SAPK pathway negatively impacts the activity of the CIP through the transcriptional induction of shared MAPK phosphatases (Fig. 1A) (12).

In this work, we provide evidence to show that Rnc1 is phosphorylated by Sty1 in multiple sites during growth and stress to elicit Rnc1 binding and destabilization of mRNA transcripts encoding several components of the SAPK pathway. This results in a reduction of Sty1 activity that ensures a precise control of cell size progression during vegetative growth and response to acute stress.

## RESULTS

### Reduced cell length at division of *rnc1Δ* cells results from enhanced SAPK activity.

We noted that cell length at division of cells of an exponentially growing mutant lacking the KH-domain RBP Rnc1 (*rnc1Δ*) is significantly reduced compared to wild-type cells (14.04 ± 0.25 versus 11.98 ± 0.29 μm, respectively) (Fig. 1B). We constructed an *rnc1Δ* mutant in a *cdc25-22* background and synchronized the cells in G_2_ by arresting the *cdc25-22* mutant at 36.5°C during 4 h (13). In this condition, *rnc1Δ* cells displayed a clear defect in polarized growth as evidenced by a reduction in cell length compared to control cells (Fig. 1C). Rnc1 activity is negatively regulated by the cell integrity pathway and its main effector the MAPK Pmk1 (5). However, cell length at division either of *pmk1Δ* cells or in a mutant strain lacking the dual-specificity phosphatase Pmp1 that dephosphorylates and inactivates Pmk1 *in vivo* (14), and whose mRNA is positively stabilized by Rnc1 (5), was similar to that of wild-type cells (Fig. 1B). Also, length at division of the double *rnc1Δ pmk1Δ* mutant was virtually identical to that of *rnc1Δ* cells (Fig. 1B). In fission yeast, the SAPK pathway and its effector MAPK Sty1 positively regulate the cell cycle at the G_2_/M transition (Fig. 1A) (7, 15, 16). While *sty1Δ* cells show an elongated cell morphology with increased length at division, those expressing the constitutively active MAPKK allele Wis1DD that hyperactivates Sty1 show a reduced cell size at division (Fig. 1B) (7). Remarkably, Sty1 deletion completely suppressed the reduced cell length at division of *rnc1Δ* cells, while the short cell length of *rnc1Δ wis1DD* cells was identical to that of the *wis1DD* mutant (Fig. 1B). In addition, basal Sty1 activity was significantly higher in exponentially growing *rnc1Δ* cells expressing a genomic C-terminal hemagglutinin (HA)-tagged version of the MAP kinase, compared to wild-type cells or a *pmk1Δ* mutant (Fig. 1D). These observations strongly suggest that the reduced cell length at division of *rnc1Δ* cells during unperturbed growth is due to enhanced basal phosphorylation of MAP kinase Sty1.

### mRNA and protein levels of positive and negative regulatory members of the SAPK pathway are increased in *rnc1Δ* cells.

**FIG 2:**
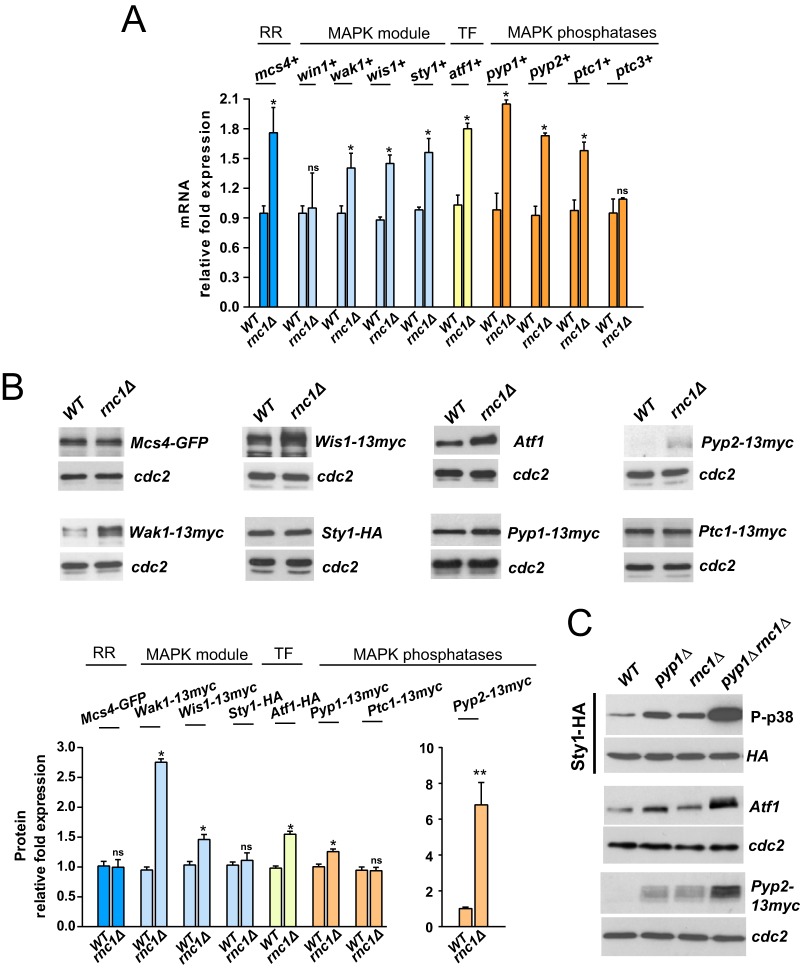
(A) mRNA levels of the indicated genes were measured by qPCR from total RNA extracted from cell samples corresponding to S. pombe wild-type and *rnc1*Δ strains growing exponentially in YES medium. Results are shown as relative fold expression (mean ± SD) from three biological repeats. *, *P < *0.05; ns, not significant, as calculated by unpaired Student’s *t* test. RR, response regulator; TF, transcription factor. (B) (Upper panel) Total extracts from growing cultures of wild-type and *rnc1*Δ strains or those expressing Mcs4-GFP, Wak1-13myc, Wis1-13myc, Sty1-HA, Pyp1-13myc, Pyp2-13myc, and Ptc1-13myc genomic fusions were resolved by SDS-PAGE, and the levels of the respective proteins were detected by incubation with anti-Atf1, anti-HA, anti-GFP, and anti-c-*myc* antibodies. Anti-Cdc2 was used as a loading control. (Lower panel) Quantification of Western blot experiments. *, *P* < 0.05; ns, not significant, as calculated by unpaired Student’s *t* test. (C) S. pombe wild-type, *pyp1Δ*, *rnc1Δ*, and *pyp1Δ rnc1Δ* cells expressing a genomic Sty1-HA6his fusion were grown in YES medium to mid-log phase. Activated and total Sty1 were detected with anti-phospho-p38 and anti-HA antibodies, respectively. Total levels of Atf1 and Pyp2-13myc fusion were determined as described for panel B.

Considering the above results, we hypothesized that Rnc1 might somehow modulate Sty1 activity by regulating mRNA levels of specific SAPK components (Fig. 1A). We employed quantitative real-time PCR (qPCR) analysis to systematically and comparatively analyze mRNA expression levels of several core members of this signaling cascade in exponentially growing *rnc1Δ* cells compared to wild-type cells. Strikingly, mRNA levels of the response regulator Mcs4; the redundant MAPKKK Wak1; the MAPKK Wis1; the MAPK Sty1; the transcription factor Atf1; and the Sty1 phosphatases Pyp1, Pyp2, and Ptc1 (7) (Fig. 1A) were significantly increased in *rnc1Δ* cells compared to wild-type cells (Fig. 2A).

Quantitative Western blot analysis of strains expressing genomic epitope-tagged fusions revealed that total protein levels of Wak1, Wis1, Atf1, Pyp1, and Pyp2, but not those of Mcs4, Sty1, and Ptc1, became also upregulated in *rnc1Δ* cells with regard to wild-type or *pmk1Δ* cells (Fig. 2B). Modification of the 3′ untranslated regions (UTRs) of the native genes introduced for expression of the corresponding proteins as genomic C-terminal epitope-tagged fusions could have reduced or inhibited Rnc1-mRNA binding in these specific genetic backgrounds. Thus, Rnc1 downregulates both mRNA and the ensuing protein levels of positive (Wak1, Wis1) and negative (Pyp1, Pyp2, and Ptc1) regulators of Sty1 phosphorylation. However, *rnc1Δ* cells show a net increase in MAPK basal phosphorylation (Fig. 1D), suggesting that Rnc1-mediated downregulation of Sty1 activators might be more biologically relevant *in vivo*. Indeed, simultaneous deletion of Pyp1 tyrosine phosphatase, which dephosphorylates Sty1 *in vivo* and whose mRNA and protein levels are enhanced in *rnc1Δ* cells (Fig. 2A and B), further increased basal Sty1 phosphorylation levels in vegetatively growing *rnc1Δ* cells (*pyp1Δ rnc1Δ* double mutant) compared to the single-mutant counterparts (Fig. 2C). The additive rise in Sty1 activity of *rnc1Δ pyp1Δ* cells was accompanied by enhanced expression of Atf1 transcription factor and Pyp2 tyrosine phosphatase protein levels compared to the single mutants (Fig. 2C). Therefore, Rnc1 prompts a decrease in mRNA levels of specific positive and negative regulators within the SAPK pathway during vegetative growth that results in a reduced Sty1 activity *in vivo*.

**FIG 3:**
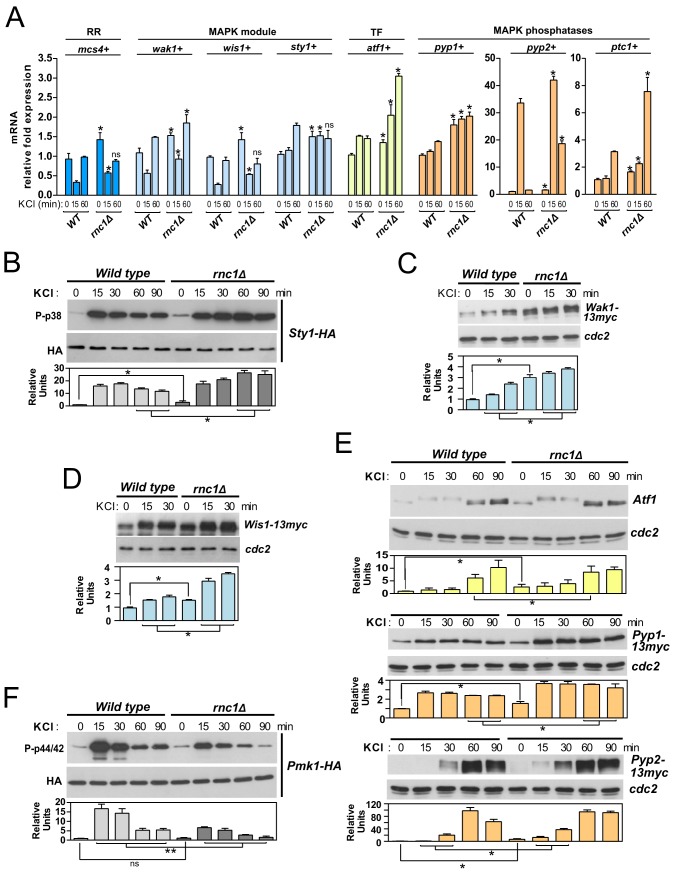
(A) mRNA levels of the indicated genes were measured by qPCR from total RNA extracted from cell samples corresponding to S. pombe wild-type and *rnc1*Δ strains growing exponentially in YES medium and treated with 0.6 M KCl for the indicated times. Results are shown as relative fold expression (mean ± SD) from three biological repeats. *, *P < *0.05; ns, not significant, for the comparisons of *rnc1*Δ cells with the corresponding incubation times of wild-type cells as calculated by unpaired Student’s *t* test. (B) S. pombe wild-type and *rnc1Δ* cells expressing a genomic Sty1-HA6his fusion were grown in YES medium to mid-log phase and treated with 0.6 M KCl for the indicated times. Activated and total Sty1 were detected with anti-phospho-p38 and anti-HA antibodies, respectively. Relative units as mean ± SD (biological triplicates) for Sty1 phosphorylation (anti-phospho-p38 blot) were determined with respect to the internal control (anti-HA blot). *, *P* < 0.05, as calculated by unpaired Student’s *t* test. (C and D) S. pombe wild-type and *rnc1Δ* cells expressing either genomic Wak1-13myc or Wis1-13myc fusions were grown in YES medium to mid-log phase and treated with 0.6 M KCl for the indicated times, and total levels of Wak1-13myc and Wis1-13myc were detected by incubation with anti-c-*myc* antibodies. Anti-Cdc2 was used as a loading control. *, *P* < 0.05, as calculated by unpaired Student’s *t* test. (E) Total extracts from growing cultures of wild-type and *rnc1*Δ strains or those expressing Pyp1-13myc or Pyp2-13myc genomic fusions, treated with 0.6 M KCl for the indicated times, were resolved by SDS-PAGE, and the levels of the respective proteins were detected by incubation with anti-Atf1 and anti-c-*myc* antibodies. Anti-Cdc2 was used as a loading control. *, *P *< 0.05, as calculated by unpaired Student’s *t* test. (F) S. pombe wild-type and *rnc1Δ* cells expressing a genomic Pmk1-HA6his fusion were grown in YES medium to mid-log phase and treated with 0.6 M KCl for the indicated times. Activated and total Pmk1 were detected with anti-phospho-p44/42 and anti-HA antibodies, respectively. **, *P* < 0.005; ns, not significant, as calculated by unpaired Student’s *t* test.

The SAPK pathway becomes activated and plays a main role in S. pombe during cell survival under multiple environmental cues, including salt stress (7). We performed a comparative qPCR analysis of mRNA expression of SAPK gene members in wild-type and *rnc1Δ* cells after 15 and 60 min following an osmotic saline stress with 0.6 M KCl. As shown in Fig. 3A, and confirming previous observations (9), mRNA expression levels of *mcs4*^+^, *wak1*^+^, and *wis1*^+^ genes decreased during saline stress in wild-type cells at early incubation times (15 min) and recovered thereafter. However, the drop in expression of the above genes was significantly less severe in salt-stressed *rnc1Δ* cells (Fig. 3A). Also, compared to wild-type cells, Rnc1 absence elicited a further increase in mRNA expression levels in genes encoding Atf1 transcription factor (*atf1*^+^), and phosphatases Pyp1, Pyp2, and Ptc1 (*pyp1*^+^, *pyp2*^+^, *ptc1*^+^), which reached its maximum after 15 min of treatment (*pyp2*^+^) or at longer incubation times (*atf1*^+^, *pyp1*^+^, *ptc1*^+^). Therefore, Rnc1 downregulates mRNA levels of positive and negative regulators of the SAPK pathway in response to stress.

The overall magnitude and dynamics of Sty1 activation during saline stress increased in the *rnc1Δ* mutant compared to wild-type cells (Fig. 3B). Although expression of *wak1*^+^ (MAPKKK) and *wis1*^+^ (MAPKK) genes becomes downregulated in wild-type cells shortly after salt stress (Fig. 3A), the respective Wak1 and Wis1 protein levels rose during this treatment, and Wis1 underwent a clear mobility shift whose origin remains unknown, as previously shown (Fig. 3C and D) (17). Again, KCl-treated *rnc1Δ* cells showed a relative increment in both Wak1 and Wis1 protein levels and mobility shift compared to wild-type cells (Fig. 3C and D). Sty1 hyperphosphorylation in salt-stressed *rnc1Δ* cells resulted in a concomitant increase in the expression levels of its downstream targets Atf1 transcription factor and the tyrosine phosphatases Pyp1 and Pyp2 (Fig. 3D). Both phosphatases, whose transcriptional induction takes place via Sty1-Atf1, are also known to dephosphorylate activated Pmk1 *in vivo* (12). We did not observe significant differences in basal Pmk1 activity between vegetatively growing wild-type cells and *rnc1Δ* cells (see Fig. S1 in the supplemental material). Importantly, Pmk1 activation in response to a salt stress was significantly lower in *rnc1Δ* cells with respect to wild-type cells (Fig. 3F), suggesting that in this mutant enhanced expression of Pyp1 and Pyp2 reinforces the inhibitory cross talk between the SAPK and the CIP signaling cascades. Hence, Rnc1-dependent downregulation of levels of mRNA encoding SAPK members warrants proper activation of Sty1 and Pmk1 MAPKs in response to stress.

10.1128/mBio.02815-19.1 FIG S1 S. pombe wild type, *sty1Δ*, and *rnc1Δ* cells expressing a genomic Pmk1-HA6his fusion were grown in YES medium to mid-log phase. Activated and total Pmk1 were detected with anti-phospho-p44/42 and anti-HA antibodies, respectively. Relative units as mean ± SD (biological triplicates) for Pmk1 phosphorylation (anti-phospho-p44/42 blot) were determined with respect to the internal control (anti-HA blot). **, *P* < 0.005; ns, not significant, as calculated by unpaired Student’s *t* test. Download FIG S1, EPS file, 1.3 MB. Copyright © 2020 Prieto-Ruiz et al. 2020 Prieto-Ruiz et al. This content is distributed under the terms of the Creative Commons Attribution 4.0 International license.

### MAPK-dependent phosphorylation of Rnc1 *in vivo* during growth and stress is strongly dependent on Sty1 function.

**FIG 4:**
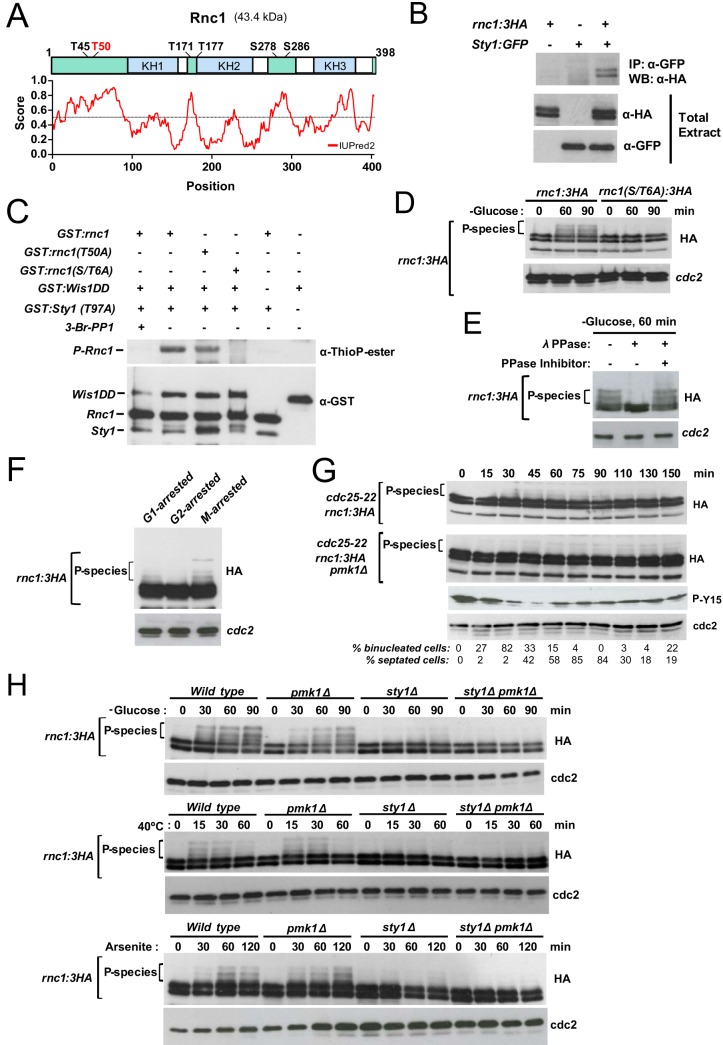
(A) Secondary structure of Rnc1. KH domains appear colored in light blue. Putative S/T MAPK-phosphosites are shown. Prediction of intrinsically disordered regions (light green boxes) with IUpred2 (https://iupred2a.elte.hu/) is shown below. (B) Coimmunoprecipitation of Rnc1-3HA and Sty-GFP genomic fusions from yeast extracts obtained from vegetatively growing cultures of the indicated genotypes. Results from a representative experiment are shown. IP, immunoprecipitation; WB, Western blot. (C) Bacterially purified GST-Rnc1, GST-Rnc1(T50A), or GST-Rnc1(S/T56A) fusions were incubated with ATP-γ-S and GST-Wis1DD (constitutively active MAPKK) and GST-Sty1-(T97A) (analog-sensitive MAP kinase), in the presence or absence of a specific kinase inhibitor (3-Br-PP1). Rnc1 thiophosphorylation was detected with anti-thioP-ester antibody. Total Wis1, Sty1, and Rnc1 levels in the reaction mixture were determined after incubation with anti-GST antibody. Results from a representative experiment are shown. (D) S. pombe cells expressing Rnc1-3HA or Rnc1(S/T6A)-3HA genomic fusions were grown in YES medium with 7% glucose, recovered by filtration, resuspended in the same medium lacking glucose, and osmotically equilibrated with 3% glycerol for the indicated times. Total and phosphorylated Rnc1 levels were determined by immunoblotting TCA-precipitated protein extracts with anti-HA antibody. Anti-Cdc2 was used as a loading control. Results from a representative experiment are shown. P-species, Rnc1-phosphorylated species. (E) Extracts from S. pombe growing cells starved for glucose for 60 min and expressing a genomic Rnc1-3HA fusion were treated with lambda phosphatase in the presence/absence of specific phosphatase inhibitor. Total and phosphorylated Rnc1 levels were determined by immunoblotting with anti-HA antibody. Anti-Cdc2 was used as a loading control. Results from a representative experiment are shown. (F) *cdc10-129* (G_1_-phase arrest), *cdc25-22* (G_2_-phase arrest), and *nda3-km311* (M-phase arrest) mutants expressing a genomic Rnc1-3HA fusion were incubated at either 36.5°C for 3.5 h (*cdc10-129* and *cdc25-22* backgrounds) or 18°C for 7 h (*nda3-km311* background). Total and phosphorylated Rnc1 levels were determined by immunoblotting with anti-HA antibody. Anti-Cdc2 was used as a loading control. Results from a representative experiment are shown. (G) Cells from *cdc25-22* and *cdc25-22 pmk1*Δ strains expressing a genomic Rnc1-3HA fusion were grown to an *A*_600_ of 0.3 at 25°C, shifted to 37°C for 3.5 h, and then released from the growth arrest by transfer back to 25°C. Aliquots were taken at the indicated time intervals, and Rnc1, Cdc2 phosphorylation at Y15, or total Cdc2 was detected by immunoblotting with anti-HA, anti-Cdc2 pY15, and anti-Cdk1/Cdc2 (PSTAIR) antibodies, respectively. Numbers at bottom show the corresponding percentages of binucleated and septated cells. Results from representative experiments are shown. (H) Wild-type, *pmk1*Δ, *sty1*Δ, and *sty1*Δ *pmk1*Δ strains expressing an Rnc1-3HA genomic fusion were grown in YES medium, resuspended in the same medium lacking glucose, and osmotically equilibrated with 3% glycerol (top panel), incubated at 40°C (middle panel), or treated with 0.5 mM sodium arsenite (bottom panel) for the indicated times. Total and phosphorylated Rnc1 levels were determined by immunoblotting of TCA-precipitated protein extracts with anti-HA antibody. Anti-Cdc2 was used as a loading control. Results from representative experiments are shown.

The cell integrity pathway MAPK Pmk1 associates with and phosphorylates Rnc1 *in vivo* at a threonine residue at position 50 located within a perfect MAPK consensus phosphosite (Fig. 4A) (5). The results obtained so far suggested that this residue might also be targeted by Sty1 *in vivo*. Coimmunoprecipitation of genomic Rnc1-3HA and Sty1-GFP fusions from yeast extracts confirmed that the two proteins associate *in vivo* (Fig. 4B). We used an *in vitro* thiophosphate assay to test whether an analog-sensitive Sty1 kinase allele, *sty1*(*T97A*), directly phosphorylates Rnc1. As shown in Fig. 4B, bacterially purified glutathione *S*-transferase (GST)–Sty1(T97A) activated by a constitutively active version of Wis1 MAPKK (GST-Wis1DD) was able to effectively thiophosphorylate a GST-Rnc1 fusion *in vitro*. This modification was dependent upon Sty1 kinase activity since it was totally inhibited in the presence of the specific analog-sensitive inhibitor 3BrPP1 (Fig. 4C). Importantly, compared to wild-type GST-Rnc1, thiophosphorylation of a GST-Rnc1(T50A) mutated version by Sty1 was somewhat reduced but not totally abrogated (Fig. 4C). Besides T50, the Rnc1 amino acid sequence has five additional putative MAPK phosphorylation sites (T45, T171, T177, S278, and S286) (Fig. 4A). Remarkably, Sty1 failed to thiophosphorylate an Rnc1 fusion where all the six S/T residues were changed to alanine [GST-Rnc1(S/T6A)] (Fig. 4C). These results suggest that while T50 is a main phosphorylation site for Sty1 within Rnc1, other phosphosites are likely targeted by this kinase *in vivo*.

We found that a genomic Rnc1-3HA fusion migrates during unperturbed vegetative growth in SDS-PAGE as a doublet that undergoes a partial mobility shift during nutritional stress in the absence of glucose (Fig. 4D). Phosphatase lambda treatment of glucose-limited cell extracts in the presence/absence of a phosphatase-specific inhibitor revealed that the Rnc1 mobility shift is due to phosphorylation (Fig. 4E). Remarkably, phosphorylated species were not detected in cells expressing an Rnc1(S/T6A)-3HA fusion in the absence of the sugar (Fig. 4D). Hence, these shifted bands represent a subset of Rnc1 MAPK-phosphorylated species and can be employed as readout to follow Rnc1 phosphorylation by MAPKs under different biological contexts. Despite their low abundance during asynchronous unperturbed growth, Rnc1 MAPK-phosphorylated species were enriched during the M phase of cell cycle as evidenced in cells arrested in an *nda3-km311* background (Fig. 4F). MAPK phosphorylation of Rnc1 was minimal during G_2_ arrest in a *cdc25-22* background, increased progressively after release during mitosis and G_1_/S phases, and decreased again as cells entered into G_2_ (Fig. 4G). Remarkably, this phosphorylation pattern was also conserved in G_2_-released *cdc25-22 pmk1Δ* cells (Fig. 4G). Although we could not follow Rnc1 phosphorylation in a *sty1Δ* background because Sty1 deletion negatively interferes with G_2_ arrest in the *cdc25-22* background (15), the above results suggest that Sty1 is mostly responsible for the cell cycle-dependent phosphorylation of Rnc1.

Similar to glucose deprivation, both Sty1 and Pmk1 become activated in S. pombe in response to other stimuli, including heat stress or the presence of arsenite (18, 19). As can be seen in Fig. 4H, MAPK-dependent phosphorylation of Rnc1 was detected with different magnitudes and dynamics in wild-type cells in response to each of the above stressors. Importantly, Sty1 absence elicited a major reduction in Rnc1 phosphorylation under all the conditions analyzed (Fig. 4H). In contrast, the impact of Pmk1 deletion on Rnc1 phosphorylation was somewhat evident in the absence of glucose, but it was virtually nonexistent under the remaining stimuli (Fig. 4H). Taken together, these results indicate that Sty1 is the main MAPK that phosphorylates Rnc1 *in vivo*, both during unperturbed growth and in response to stress.

### Rnc1 binds to mRNAs encoding SAPK components to promote their destabilization *in vivo*.

**FIG 5:**
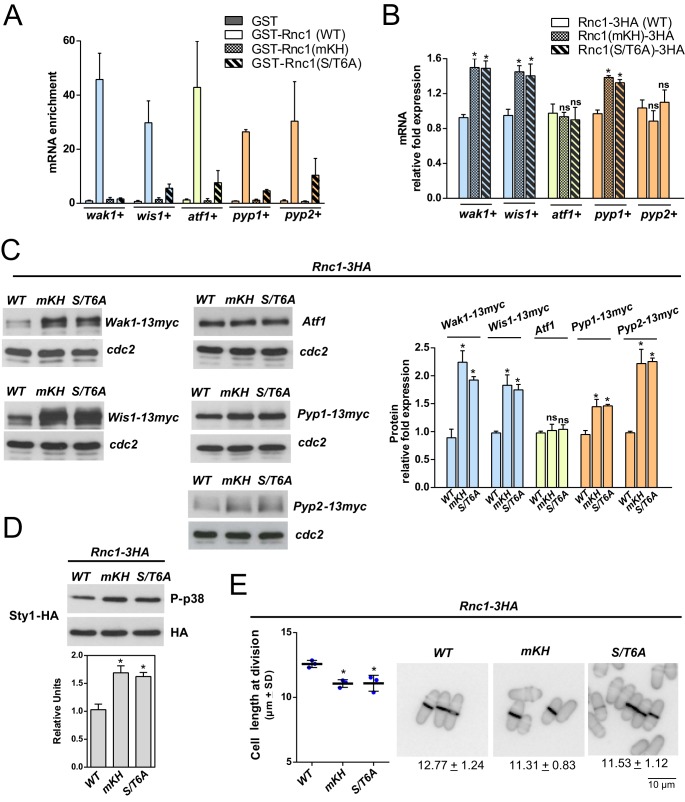
(A) GST, GST-Rnc1, GST-Rnc1(mKH), and GST-Rnc1(S/T6A) fusions purified from S. pombe cultures were separately incubated with total RNA, and after extensive washes, the RNA-binding ability of Rnc1 with respect to the indicated transcripts was measured by RT-qPCR and normalized with *leu*^+^ mRNA. (B) mRNA levels of the indicated genes were measured by qPCR from total RNA extracted from cell samples corresponding to S. pombe cells growing exponentially in YES medium and expressing either Rnc1-3HA (wild type), Rnc1(mKH)-3HA, or Rnc1(S/T6A)-3HA genomic fusions. Results are shown as relative fold expression (mean ± SD) from three biological repeats. *, *P < *0.05; ns, not significant, as calculated by unpaired Student’s *t* test. (C) (Left) Total extracts from growing cultures of strains coexpressing either Rnc1-3HA (wild type), Rnc1(mKH)-3HA, or Rnc1(S/T6A)-3HA with Wak1-13myc, Wis1-13myc, Pyp1-13myc, or Pyp2-13myc genomic fusions were resolved by SDS-PAGE, and the levels of the indicated proteins were detected by incubation with anti-Atf1 and anti-c-*myc* antibodies. Anti-Cdc2 was used as a loading control. (Right) Quantification of Western blot experiments. *, *P* < 0.05; ns, not significant, as calculated by unpaired Student’s *t* test. (D) Rnc1-3HA (wild type), Rnc1(mKH)-3HA, and Rnc1(S/T6A)-3HA cells expressing genomic Sty1-HA6his fusions were grown in YES medium to mid-log phase. Activated and total Sty1 were detected with anti-phospho-p38 and anti-HA antibodies, respectively. Relative units as mean ± SD (biological triplicates) for Sty1 phosphorylation (anti-phospho-p38 blot) were determined with respect to the internal control (anti-HA blot). *, *P* < 0.05, as calculated by unpaired Student’s *t* test. (E) Cell lengths at division of S. pombe Rnc1-3HA (wild type), Rnc1(mKH)-3HA, and Rnc1(S/T6A)-3HA cells growing exponentially in YES medium are presented as scatter plots showing the average values ± SD (number of independent biological replicates = 3; number of cells ≥ 200/strain). Significant differences were assessed by Tukey’s test following one-way ANOVA for the comparisons with respective values of wild-type cells. *, *P < *0.05. Cell morphology of each strain was analyzed by fluorescence microscopy after staining with calcofluor white. Bar, 10 μm.

RBPs recognize and bind target mRNA molecules in a sequence-dependent as well as an independent manner and promote either their degradation or their stabilization (20). It has been shown elsewhere that a double mutation in the hallmark GxxG loop (GxxG to CDDG) impairs nucleic acid binding of KH domains without compromising their stability (2). Therefore, we constructed a mutant Rnc1 version where the two residues within each of the three conserved GXXG loops of the respective KH domains are replaced by aspartic acid [Rnc1(m3KH)]. We expressed in S. pombe N-terminal GST-fused versions of wild-type and mRNA-binding-defective Rnc1 (m3KH). GST alone and the purified fusions were then mixed with fission yeast total RNA, and after incubation with glutathione-Sepharose beads and extensive washing, the bound mRNAs were subjected to RT-qPCR analysis to quantify those encoding SAPK components. As shown in Fig. 5A, *wak1*^+^, *wis1*^+^, *atf1*^+^, *pyp1*^+^, and *pyp2*^+^ mRNAs copurified and were selectively enriched to different ratios (10× to 60×) with wild-type GST-Rnc1 compared to the GST-Rnc1(m3KH) mutated version, suggesting that KH domains mediate Rnc1 binding to mRNAs of SAPK components *in vitro*. Compared to control Rnc1-3HA cells, *wak1*^+^, *wis1*^+^, and *pyp1*^+^ mRNA levels, but not those of *atf1*^+^ and *pyp2*^+^, increased significantly in growing cells expressing a genomic Rnc1 fusion lacking mRNA binding ability [Rnc1(m3KH)-3HA] (Fig. 5B). Wak1, Wis1, and Pyp1 protein levels were also higher in Rnc1(m3KH)-3HA cells, whereas Atf1 levels remained unchanged (Fig. 5C). Although *pyp2*^+^ mRNA levels were almost identical in control and m3KH cells, Pyp2 protein levels increased ∼2 times in the mutant background (Fig. 5C), but they were of a lower magnitude than that in *rnc1Δ* versus wild-type cells (∼8 to 9 times) (Fig. 2C). It may be possible that Rnc1-m3KH is still able to bind *atf1*^+^ and *pyp2*^+^ mRNAs to some extent. Alternatively, the Rnc1-3HA fusion used in these constructs might be not fully functional. In support for this hypothesis, both basal Sty1 phosphorylation and cell length at division were slightly increased and reduced, respectively, in Rnc1-3HA cells with respect to the parental strain expressing wild-type (unfused) Rnc1 (Fig. S2). In any case, and like *rnc1Δ* cells, Rnc1(m3KH)-3HA cells displayed enhanced basal Sty1 activity (Fig. 5D) and decreased cell length at division (Fig. 5E) with respect to the isogenic wild-type counterpart.

10.1128/mBio.02815-19.2 FIG S2 (A) Cell length at division of S. pombe wild-type and Rnc1-3HA cells growing exponentially in YES medium showing the average values ± SD (number of independent biological replicates = 3). Cell morphology of each strain was analyzed by fluorescence microscopy after staining with calcofluor white. Bar, 10 μm. (B) S. pombe wild-type and Rnc1-3HA cells expressing genomic Sty1-HA6his fusions were grown in YES medium to mid-log phase. Activated and total Sty1 were detected with anti-phospho-p38 and anti-HA antibodies, respectively. Relative units as mean ± SD (biological triplicates) for Sty1 phosphorylation (anti-phospho-p38 blot) were determined with respect to the internal control (anti-HA blot). **, *P* < 0.005, as calculated by unpaired Student’s *t* test. Download FIG S2, EPS file, 1.7 MB. Copyright © 2020 Prieto-Ruiz et al. 2020 Prieto-Ruiz et al. This content is distributed under the terms of the Creative Commons Attribution 4.0 International license.

**FIG 6:**
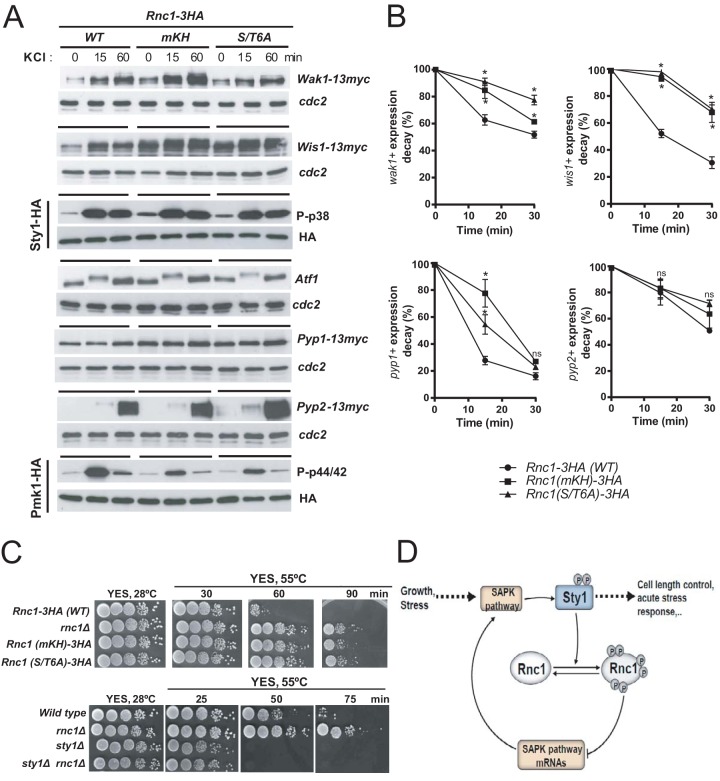
(A) Total extracts from growing cultures of Rnc1-3HA (wild-type), Rnc1(mKH)-3HA, and Rnc1(S/T6A)-3HA cells growing exponentially and expressing either Wak1-13myc, Wis1-13myc, Sty1-HA6his, Pyp1-13myc, Pyp2-13myc, or Pmk1-HA6his genomic fusions were treated with 0.6 M KCl for the indicated times. Total levels of Wak1, Wis1, Atf1, Pyp1, and Pyp2 were detected by incubation with anti-Atf1 and anti-c-*myc* antibodies. Anti-Cdc2 was used as a loading control. Activated and total Sty1 were detected with anti-phospho-p38 and anti-HA antibodies, respectively. Activated and total Pmk1 were detected with anti-phospho-p44/42 and anti-HA antibodies, respectively. Results from representative experiments are shown. (B) Percentages of decay in the expression levels of *wak1*^+^, *wis1*^+^, *pyp1*^+^, and *pyp2*^+^ mRNAs with respect to 28S RNA (no decay during the experiment) were measured by qPCR from S. pombe cultures expressing either Rnc1-3HA (wild type), Rnc1(mKH)-3HA, or Rnc1(S/T6A)-3HA genomic fusions and treated for the indicated times with 1,10-phenanthroline to block transcription. Results are shown as relative fold expression (mean ± SD) from three biological repeats. *, *P < *0.05; ns, not significant, as calculated by unpaired Student’s *t* test. (C) Decimal dilutions of strains of the indicated genotypes were spotted on YES solid plates and incubated in an oven at 55°C for the indicated times. The plates were then removed from the oven, incubated at 30°C for 3 days, and photographed. Representative experiments are shown. (D) Cross-regulatory interactions between Rnc1 and the stress-activated MAPK signaling pathway (SAPK) in fission yeast. For specific details, please see text.

Compared to control cells, Sty1 activation and Atf1 protein levels did not rise significantly in response to a saline osmotic stress in Rnc1-m3KH cells lacking mRNA binding ability (Fig. 6A). In contrast, they showed a reproducible increase in protein expression of MAPKKK Wak1, MAPKK Wis1, and tyrosine phosphatases Pyp1 and Pyp2 relative to control cells (Fig. 6A) and a marked reduction in Pmk1 activation when subjected to an identical treatment (Fig. 6A). Altogether, these findings suggest that downregulation of Sty1 activity by Rnc1 relies on its ability to bind specific mRNAs (*wak1*^+^, *wis1*^+^, *pyp1*^+^, *pyp2*^+^) encoding components of the SAPK pathway. However, if this hypothesis is correct, Rnc1 binding should decrease the stability of these mRNAs. To explore this possibility, exponentially growing cultures of Rnc1-3HA (wild type) and Rnc1(m3KH)-3HA cells were treated with 250 μg/ml of 1,10-phenanthroline to block transcription (11); total RNAs were extracted at different times, and the decay in mRNA levels of SAPK transcripts was determined by RT-qPCR analysis (see Materials and Methods). As shown in Fig. 5D, the half-lives of *wak1*^+^, *pyp1*^+^, *wis1*^+^, and *pyp2*^+^ mRNAs were significantly higher in Rnc1(m3KH)-3HA cells than wild-type cells, suggesting that Rnc1 binding promotes their destabilization *in vivo*.

### MAPK-dependent phosphorylation of Rnc1 *in vivo* is essential to negatively regulate Sty1 activity during control of cell length at division and adaptive response to acute stress.

The observation that Sty1 phosphorylates Rnc1 *in vivo* at multiple S/T residues during unperturbed growth and stress (Fig. 4) prompted us to further evaluate the impact of this posttranslational modification on its function as an RBP. A nonphosphorylatable GST-Rnc1 fusion [GST-Rnc1(S/T6A)] expressed in fission yeast was several times less effective than the wild type (GST-Rnc1) in binding *wak1*^+^, *wis1*^+^, *atf1*^+^, *pyp1*^+^, and *pyp2*^+^ mRNAs *in vitro* (Fig. 5A). Moreover, S. pombe cells expressing a genomic nonphosphorylatable HA-fused Rnc1 mutant version [Rnc1(S/T6A)-3HA] were phenotypically similar to those defective in mRNA binding [Rnc1(m3KH)-3HA], as they showed increased mRNA levels (Fig. 5B) and half-lives (Fig. 6B) of *wak1*^+^, *wis1*^+^, and *pyp1*^+^ and enhanced expression of Wak1, Wis1, and Pyp1 proteins during unperturbed growth (Fig. 5C) and in response to stress (Fig. 6A). These phenotypes were also accompanied by a net increase in basal Sty1 activity (Fig. 5D), and reduced cell length at division (Fig. 5E). Therefore, Sty1-dependent phosphorylation impairs Rnc1 binding and destabilization of mRNAs encoding SAPK members and the ensuing reduction in MAPK activity to control cell length at division during growth and stress.

In S. pombe, the SAPK pathway and its core member Sty1 MAPK control multiple cellular events, including cell survival in response to environmental cues (7). Many of these adaptive responses are executed through a transcriptional program involving expression of Atf1-dependent genes (9). Consequently, *sty1Δ* cells show strong growth sensitivity when facing different stressors, including high temperature, saline stress (KCl, NaCl), oxidative stress (hydrogen peroxide), or caffeine (7, 21, 22) (Fig. S3). The observation that mutants lacking Rnc1 display enhanced Sty1 activity and Atf1 expression suggested that this situation might favor cellular adaptation and survival in response to environmental stress. However, growth sensitivity of *rnc1Δ* cells was virtually identical to that of wild-type cells in response to the above treatments (Fig. S3), suggesting that Rnc1 does not play a significant role in the adaptive cellular response to stress. Remarkably, we found that, compared to control cells, *rnc1Δ* cells exhibited a significant increase in growth recovery after being subjected to an acute thermal stress (55°C) during 60 to 90 min, and this phenotype was shared by both Rnc1(m3KH)-3HA and Rnc1(S/T6A)-3HA cells (Fig. 6C). Moreover, Sty1 absence completely suppressed the enhanced growth recovery phenotype of *rnc1Δ* cells (Fig. 6C). Taken together, these results suggest that Sty1-dependent phosphorylation triggers Rnc1-mRNA binding to negatively regulate S. pombe cell growth and survival in response to acute stress (Fig. 6D).

10.1128/mBio.02815-19.3 FIG S3 Decimal dilutions of strains of the indicated genotypes were spotted on YES solid plates with the indicated compounds, incubated at either 28 or 36°C for 3 days, and then photographed. Representative experiments are shown. Download FIG S3, EPS file, 2.5 MB. Copyright © 2020 Prieto-Ruiz et al. 2020 Prieto-Ruiz et al. This content is distributed under the terms of the Creative Commons Attribution 4.0 International license.

## DISCUSSION

In this work, we show that the KH-domain RBP Rnc1 downregulates SAPK function in S. pombe during control of cell length at division and the adaptive response to acute stress (Fig. 6D). This assumption is based in the finding that, compared to wild-type cells, *rnc1Δ* cells display increased basal Sty1 activity that results in a reduction in length at division and enhanced growth recovery after acute thermal stress. Importantly, Rnc1 negative control of SAPK function is strictly dependent on its ability to bind mRNAs encoding both activators (Wak1 MAPKKK and Wis1 MAPKK) and negative regulators (Atf1 transcription factor and Pyp1 and Pyp2 tyrosine phosphatases) of Sty1 phosphorylation through its KH domains. Consequently, cells expressing a KH-domain mutated version of the RBP unable to bind mRNA (Rnc1-m3KH) phenocopied *rnc1Δ* cells and showed increased Sty1 activity, reduced cell length at division, and enhanced tolerance to heat shock. As a whole, our observations depict a new role for Rnc1 as a negative modulator of SAPK function in fission yeast (Fig. 6D).

This novel mechanism seems unrelated to Rnc1 downregulation of CIP signaling, which relies on its ability to bind and stabilize *pmp1*^+^ mRNA encoding the dual-specificity phosphatase Pmp1, which dephosphorylates and inactivates Pmk1 *in vivo* (5). Indeed, our results suggest that Rnc1 binding prompts instead the destabilization of specific mRNAs encoding core upstream and downstream components of the SAPK cascade, including at least *wak1*^+^, *wis1*^+^, and *pyp1*^+^. This assumption is sustained by the increased expression and half-lives of these mRNAs found in Rnc1-m3KH cells during unperturbed growth relative to wild-type cells. Signal transmission to Wis1 during growth and saline stress is mediated by a Wak1-Win1 MAPKKK heteromer complex stabilized by Mcs4 (17). We thus propose that in *rnc1Δ* cells the enhanced expression of *wak1*^+^ and *wis1*^+^ mRNAs, which results in increased availability of Wak1 and Wis1 proteins, allows for a more efficient downstream transmission from this module and the ensuing activation of Sty1 MAPK. Indeed, the RBP-mediated negative-control *wak1*^+^ and *wis1*^+^ mRNAs seems more biologically relevant than downregulation of *atf1*^+^, *pyp1*^+^, and *pyp2*^+^ mRNAs, as *rnc1Δ* cells display a net increase in MAPK activity. Moreover, although enhanced expression of *mcs4*^+^, *sty1*^+^, and *ptc1*^+^ mRNAs in *rnc1Δ* cells was not accompanied by a parallel increase in the respective protein levels expressed as C-terminally tagged fusions, the possibility that Rnc1-dependent downregulation of Mcs4, Sty1, and Ptc1 expression also impinges on SAPK signaling cannot be ruled out. Previous global transcriptome sequencing (RNA-seq) analysis showed that the number of upregulated genes in vegetatively growing *rnc1Δ* cells (including *pyp1*^+^, which has been confirmed in this work to be a direct target for Rnc1) is larger than those being downregulated (4). Thus, it seems highly likely that the role of Rnc1 as a negative regulator of mRNA half-life/stability is extended to mRNAs other than those encoding SAPK components.

Increased mRNA expression of *wak1*^+^, *wis1*^+^, and *pyp1*^+^ genes in *rnc1Δ* and Rnc1-m3KH cells resulted in the ensuing rise in Wak1, Wis1, and Pyp1 protein levels not only during vegetative growth but also in response to stress, suggesting that Rnc1 binding to the those mRNAs regulates SAPK function in response to a variety of environmental cues. In response to Sty1 activation, Atf1 transcription factor elicits expression of many CESR genes, including *pyp1*^+^ and *pyp2*^+^ (9). In this context, it could be possible that the enhanced levels of Atf1 present in *rnc1Δ* cells could account for the increased expression of the remaining SAPK members described in this work. However, this possibility seems highly unlikely for two main reasons. First, mRNA and protein expression levels of *wak1*^+^, *wis1*^+^, *pyp1*^+^, and *pyp2*^+^ significantly increased in cells expressing an Rnc1 mutant version unable to bind mRNAs [Rnc1(m3KH)-3HA] with respect to wild-type cells. Importantly, Atf1 levels did not change in this background (Fig. 5B and C). Second, *wak1*^+^, *wis1*^+^, *pyp1*^+^, and *pyp2*^+^ mRNAs copurified and became selectively enriched with wild-type Rnc1 (Fig. 5A), and their half-lives increased in the absence of Rnc1 function (Fig. 6B). Therefore, Rnc1-mediated downregulation of mRNAs encoding the above SAPK components involves direct binding and destabilization by the RBP and is independent of the altered expression pattern of Atf1.

While Pmk1 is a specific substrate for Pmp1 phosphatase, Pyp1 and Pyp2 dephosphorylate both Sty1 and Pmk1 *in vivo* during growth and stress (12). The finding that the magnitude of Pmk1 activation during an osmotic saline stress is strongly reduced in the *rnc1Δ* mutant compared to wild-type cells further confirms the role of Rnc1 as a negative regulator of SAPK signaling and Pyp1/2 expression. It also shows its relevance as an additional player whereby SAPK may decrease CIP signaling in response to environmental stimuli.

Posttranslational modification by phosphorylation has a major influence on RBP function and affinity toward their targets, with the consequent positive or negative impact on mRNA stability, turnover, and translation efficiency (23, 24). *In vivo* Rnc1 phosphorylation by Pmk1 at a putative MAPK phosphosite located at T50 enhances RBP binding and stabilization of Pmp1 mRNA (5). However, in this work we show that Sty1 associates *in vivo* and phosphorylates Rnc1 not only at T50 but at additional MAPK phosphosites (T45, T171, T177, S278, and/or S286), whose precise identity remains to be determined. During unperturbed growth, a very small fraction of the total Rnc1 protein becomes phosphorylated at these sites in wild-type cells, particularly during the M and G_1_/S phases of the cell cycle. However, Rnc1 phosphorylation is much more evident when cells are subjected to treatments that activate either Sty1, like arsenite (25), or both Sty1 and Pmk1, like glucose deprivation or thermal stress (7, 18, 26). Remarkably, enhanced MAPK-dependent phosphorylation of Rnc1 was mostly absent in *sty1Δ* cells but not in *pmk1Δ* cells during vegetative growth and in response to the above stresses. These results strongly suggest that Sty1 is mainly responsible for Rnc1 phosphorylation during growth and in response to environmental cues. Moreover, the finding that the phenotypes of Rnc1-S/T6A cells lacking phosphorylatable MAPK sites (reduced cell length at division, enhanced Sty1 activity and activation under stress, and increased cell recovery after acute thermal shock) mimic those of cells expressing the mutant version unable to bind mRNA (Rnc1-m3KH) strongly suggests that Sty1-dependent phosphorylation triggers Rnc1 for proper binding and destabilization of several of its mRNA substrates (*wis1*^+^, *pyp1*^+^).

How can phosphorylated Rnc1 promote either mRNA stabilization or destabilization? An attractive possibility is that alternative phosphorylation of Rnc1 by Sty1 and/or Pmk1 at one or several S/TP sites might trigger its function as an mRNA stabilizer or destabilizer depending on the environmental context. Protein-RNA interactions were initially thought to be mostly mediated by canonical RNA-binding regions, like KH domains, that form stable secondary and tertiary structures. However, recent studies including proteome-wide data have revealed unexpected roles for intrinsically disordered protein regions in RNA binding (27). Interestingly, all of the Rnc1 MAPK phosphorylation sites (T50, T45, T171, T177, S278, and S286) are excluded from KH domains and lie within predicted intrinsically disordered regions of the protein (Fig. 4A). Alternative phosphorylation at these sites might thus elicit major changes in Rnc1 conformation and affect its ability to bind mRNAs with different affinity. The stabilizer/destabilizer role for Rnc1 might also be imposed by specific structural features of its target mRNAs. Rnc1 binds to several UCAU repeats in the 3′ UTR of *pmp1*^+^ mRNA, while mutation at these sequences impedes Rnc1 binding and prompts mRNA destabilization (5, 6). These repeats belong to the consensus YCAY RNA-binding element that is bound by KH-domain RBPs like the mammalian onconeural antigen Nova-1 (28). However, in contrast to the high number of UCAU motifs found in the long *pmp1*^+^ 3′ UTR, they have a scarce presence in the shorter *wak1*^+^ (0), *wis1*^+^ (1), *atf1*^+^ (2), *pyp1*^+^ (0), and *pyp2*^+^ (3) 3′ UTRs (Fig. S4). In addition, the possibility that Rnc1 binds to those mRNAs via UCAU motifs located at their open reading frames (ORFs) or 5′ UTRs, or through other unknown motifs, cannot be discarded.

10.1128/mBio.02815-19.4 FIG S4 UCAU motifs present at the 3′ UTR sequences corresponding to *wak1*^+^, *wis1*^+^, *atf1*^+^, *pyp1*^+^, and *pyp2*^+^ mRNAs are marked in yellow. Download FIG S4, EPS file, 2.1 MB. Copyright © 2020 Prieto-Ruiz et al. 2020 Prieto-Ruiz et al. This content is distributed under the terms of the Creative Commons Attribution 4.0 International license.

From a biological perspective, the existence of a shared control of Rnc1 function by two MAP kinases (Sty1 and Pmk1) should somehow be expected when considering that both the SAPK pathway and CIP cross talk extensively in S. pombe and become activated by a similar range of stimuli (7). Therefore, alternative Rnc1 phosphorylation by both SAPK and CIP signaling cascades might allow for exquisite differential regulation of their biological functions during unperturbed growth and in response to changing environmental conditions.

## MATERIALS AND METHODS

### Strains, growth conditions, and reagents.

The S. pombe strains used in this work are listed in Table S1 in the supplemental material. They were routinely grown with shaking at 28 or 30°C in rich (yeast extract plus supplements [YES]) or minimal (Edinburgh minimal medium [EMM2]) medium with 2% glucose and supplemented with adenine, leucine, histidine, or uracil (100 mg/liter; Sigma-Aldrich) (13). In stress experiments, log-phase cultures (OD_600_ of 0.5; ∼10^6^ cells/ml) were either incubated at 40°C (heat shock) or supplemented with KCl (Sigma-Aldrich) or sodium arsenite (Sigma-Aldrich). In glucose starvation experiments, cells grown in YES medium with 7% glucose were recovered by filtration, resuspended in the same medium lacking glucose, and osmotically equilibrated with 3% glycerol. At different times, the cells from 50 ml of culture were harvested by centrifugation at 4°C and washed with cold PBS buffer, and the yeast pellets were immediately frozen in liquid nitrogen for further analysis. Transformants expressing GST-fused Rnc1 constructs from pREP3X-based plasmids were grown in liquid EMM2 medium with thiamine (5 mg/liter) and transferred to EMM2 lacking thiamine for 24 h.

10.1128/mBio.02815-19.5 TABLE S1 S. pombe strains used in this study. Download Table S1, DOCX file, 0.02 MB. Copyright © 2020 Prieto-Ruiz et al. 2020 Prieto-Ruiz et al. This content is distributed under the terms of the Creative Commons Attribution 4.0 International license.

### Gene disruption, epitope tagging, site-directed mutagenesis, and expression of GST-tagged Rnc1 fusions.

An S. pombe *rnc1*^+^ null mutant was obtained by ORF deletion and replacement with the G418 (*kanR*) cassette by PCR-mediated strategy using plasmid pFA6a-*kanMX6* (29) and the oligonucleotides Rnc1D-FWD and Rnc1D-REV (Table S2). Plasmid pFA6a-3HA-*kanMX6* and the oligonucleotides Rnc1-CT-FWD and Rnc1-CT-REV were employed to obtain a genomic C-terminally Rnc1-3HA-tagged version. Strains expressing different genomic fusions in multiple genetic backgrounds were constructed either by transformation or after random spore analysis of appropriate crosses in sporulation agar (SPA) medium.

10.1128/mBio.02815-19.6 TABLE S2 Oligonucleotides and DNA fragments used in this study. Download Table S2, DOCX file, 0.02 MB. Copyright © 2020 Prieto-Ruiz et al. 2020 Prieto-Ruiz et al. This content is distributed under the terms of the Creative Commons Attribution 4.0 International license.

To construct the template plasmid pTA-Rnc1:HA, the *rnc1*^+^ C-terminally HA-tagged ORF plus regulatory sequences, the *kanMX6* cassette, and 3′ UTR were amplified by PCR using genomic DNA from Rnc1-3HA cells as the template and the 5′ oligonucleotide Rnc1-FWD, which hybridizes −421 to 391 bp upstream of the *rnc1*^+^ start codon, and the 3′ oligonucleotide Rnc1-REV, which hybridizes +788 to +818 bp downstream of the *rnc1*^+^ stop codon. The PCR fragment was cloned into plasmid pCR2.1 using the TA cloning kit (Thermo Fisher Scientific) and confirmed by sequencing. The Rnc1:HA (T50A) mutant was obtained by one-step site-directed mutagenesis PCR using plasmid pTA-Rnc1:HA as a template and the correspondent mutagenic oligonucleotide pairs Rnc1-T50A-FWD and Rnc1-T50A-REV (30). To obtain plasmids pTA-Rnc1(K110D, A111D, R196D, N197D, R338D, G339D):HA [synonymous with Rnc1(mKH):HA; Rnc1 mutated at the 3 KH mRNA binding domains] and pTA-Rnc1(T45A, T50A, T171A, T177A, S278A, S286A):HA [synonymous with Rnc1(S/T6A):HA; Rnc1 mutant lacking MAPK phosphosites], plasmid pTA-Rnc1:HA was digested with SacI and PacI, and the released *rnc1*^+^ ORF fragment was replaced with synthesized DNA fragments including the indicated mutations (GenParts; GenScript) and digested with SacI and PacI. The above plasmids were used as PCR templates to obtain the corresponding DNA fragments, which were transformed into wild-type strain MM1. G418-resistant transformants were obtained, and the correct integration of the respective genomic wild-type (Rnc1-3HA) and mutated [Rnc1(T50A)-3HA, Rnc1(mKH)-3HA, and Rnc1(S/T6A)-3HA] fusions was verified by both PCR and Western blot analysis. Incorporation of the mutagenized residues was confirmed by sequencing.

Bacterially expressed GST-Rnc1, GST-Rnc1(T50A), GST-Rnc1(mKH), and GST-Rnc1(S/T6A) fusions were obtained by PCR employing pTA-Rnc1:HA, pTA-Rnc1(T50A):HA, pTA-Rnc1(mKH):HA, and pTA-Rnc1(S/T6A):HA plasmids as the templates, respectively, and the oligonucleotides GSTRnc1-FWD-BamHI and GSTRnc1-REV-XbaI. The PCR products were then digested with BamHI and XbaI and cloned into plasmid pGEX-KG. To express the GST-Rnc1 fusions in S. pombe under the control of the strong version of the thiamine (B_1_)-repressible promoter from pREP3X expression plasmid (31), wild-type and mutagenized Rnc1 constructs were by amplified by PCR employing plasmids pTA-Rnc1:HA, pTA-Rnc1(T50A):HA, pTA-Rnc1(mKH):HA, and pTA-Rnc1(S/T6A):HA plasmids as the templates and the oligonucleotide pair GST-FWD-XhoI and GSTRnc1-REV-SmaI. PCR fragments were digested with XhoI and SmaI and cloned into pREP3X. GST ORF (negative control) was also cloned into pREP3X by employing pGEX-KG plasmid as the template and the oligonucleotides GST-FWD-XhoI and GST-REV-BamHI. They were separately transformed into S. pombe, and *leu1*^+^ transformants were selected in EMM2 medium plus thiamine.

### cDNA synthesis and qPCR.

S. pombe wild-type and mutant strains were grown in YES medium to a final OD_600_ of 0.5 (∼10^6^ cells/ml). Total RNAs were purified using the RNeasy minikit (Qiagen), treated with DNase (Invitrogen), and quantitated using a NanoDrop 100 spectrophotometer (Thermo Scientific). Total RNAs (1 μg) were reverse transcribed into cDNA with the iScript reverse transcription supermix (Bio-Rad). Quantitative real-time PCRs (qPCRs) were performed using the iTaq Universal SYBR green Supermix and a CFX96 real‐time PCR system (Bio‐Rad Laboratories, CA, USA). Relative gene expression was quantified based on the threshold cycle (2^−ΔΔ^*^CT^*) method and normalized using *leu1*^+^ mRNA or 28S rRNA expression in each sample. The list of gene-specific primers for qPCR is indicated in Table S2.

### mRNA-Rnc1 binding assay.

RNA-protein binding assays were carried out by following the method described by Satoh et al. (8) with slight modifications. Exponentially growing cells (4 × 10^8^ total cells) expressing N-terminally glutathione *S*‐transferase (GST)-tagged wild-type or mutated Rnc1 proteins were disrupted in 500 μl extraction buffer (30 mM Tris HCl, pH 8, 1% Triton X-100, 2 mM EDTA, 1 mM dithiothreitol [DTT], plus protease and phosphatase inhibitor cocktails [obtained from Sigma-Aldrich and Roche Molecular Biochemicals, respectively]). Glutathione-Sepharose 4B (GE Healthcare, USA) was added to the cleared extracts, which were incubated for 2 h at 4°C. Sepharose was washed seven times in wash buffer (30 mM Tris HCl, pH 8, 1% Triton X-100, 2 mM EDTA, 1 mM DTT, 3 M NaCl plus phosphatase inhibitor cocktail) and two times in binding buffer (30 mM Tris HCl, pH 8, 1% Triton X-100, and 1 mM DTT). Fission yeast total RNA (100 μg) and 100 U ml^−1^ SUPERase RNase inhibitor (Invitrogen) were added to the washed Sepharose containing equivalent amounts of the purified GST-Rnc1 fusion and incubated for 2 h at 4°C. After two washes in binding buffer, the RNA bound to Sepharose was extracted with the RNeasy minikit (Qiagen, Germany). cDNA synthesis (10 μl RNA) and qPCR were performed as described above.

### Determination of mRNA stability.

Cells were grown at 28°C in YES medium to mid-log phase (OD_660_ of 0.4), and then 1,10-phenanthroline dissolved in 100% ethanol (Sigma-Aldrich) was added to cultures to a final concentration of 250 μg ml^−1^ to inhibit transcription (11). The cells from 20 ml of culture (10^8^ total cells) were harvested, and total RNA was extracted at the indicated time points. cDNA synthesis and determination of the relative mRNA expression levels by qPCR were performed as described above.

### Detection and quantification of total and activated Pmk1 and Sty1 levels.

Preparation of cell extracts, affinity chromatography purification of HA-tagged Pmk1 or Sty1 with Ni^2+^-NTA agarose beads (Qiagen), and SDS-PAGE were performed as described previously (32). This approach strongly reduces the potential inaccuracy in the detection of both total and phosphorylated MAPKs. Dual phosphorylation in either Pmk1 or Sty1 was detected by employing rabbit polyclonal anti-phospho-p44/42 (Cell Signaling) or rabbit monoclonal anti-phospho-p38 (Cell Signaling) antibody, respectively. Total Pmk1 or Sty1 was detected with mouse monoclonal anti-HA antibody (12CA5; Roche Molecular Biochemicals). Immunoreactive bands were revealed with anti-rabbit or anti-mouse horseradish peroxidase (HRP)-conjugated secondary antibodies (Sigma-Aldrich) and the ECL system (GE Healthcare).

### Detection of Rnc1.

Cells from yeast cultures were fixed, and total protein extracts were prepared by precipitation with trichloroacetic acid (TCA) as previously described (33). Proteins were resolved in 10% SDS-PAGE gels and transferred to Hybond-ECL membranes. Rnc1-3HA fusions were detected by employing a mouse monoclonal anti-HA antibody (12CA5; Roche Molecular Biochemicals). Rabbit monoclonal anti-PSTAIR (anti-Cdc2; Sigma-Aldrich) was used for a loading control. Immunoreactive bands were revealed with anti-rabbit or anti-mouse HRP-conjugated secondary antibodies (Sigma) and the ECL system (GE Healthcare).

### Detection and quantification of Atf1 and Mcs4-, Wak1-, Wis1-, Pyp1-, Pyp2-, and Ptc1-tagged fusions.

Cell extracts were prepared using buffer IP (50 mM Tris-HCl, pH 7.5, 5 mM EDTA, 150 mM NaCl, 1 mM β-mercaptoethanol, 10% glycerol, 0.1 mM sodium orthovanadate, 1% Triton X-100, and protease inhibitors) and resolved on 8, 10, or 12% SDS-PAGE gels depending on the size of the fused protein. S. pombe Atf1 was detected with a mouse monoclonal antibody (ATF1 2A9/8) from Abcam (ab18123). Mouse polyclonal anti-GFP (Roche) was employed to detect Mcs4-GFP. Wak1-13myc, Wis1-13myc, Pyp1-13myc, Pyp2-13myc, and Ptc1-13myc fusions were detected with a mouse monoclonal anti-c-*myc* antibody (clone 9E10; Roche Molecular Biochemicals). Rabbit monoclonal anti-PSTAIR (anti-Cdc2; Sigma Chemical) was used for a loading control. Immunoreactive bands were revealed with anti-rabbit or anti-mouse HRP-conjugated secondary antibodies (Sigma-Aldrich) and the ECL system (GE Healthcare).

### Coimmunoprecipitation.

Whole-cell extracts from the appropriate strains were prepared in lysis buffer (20 mM Tris-HCl, pH 8.0, 2 mM EDTA, 100 mM NaCl, and 0.5% NP-40 and containing a protease inhibitor cocktail [Sigma-Aldrich]). Cell extracts (3 mg) were incubated with Dynabeads protein G (Novex by Life Technology) bound to polyclonal anti-GFP antibody (Roche) for 2 h at 4°C. The beads were washed four times with lysis buffer and resuspended in sample buffer, and the immunoprecipitates were then analyzed by Western blotting with either anti-GFP or anti-HA (12CA5; Roche Molecular Biochemicals) antibodies, followed by immunodetection with anti-mouse HRP-conjugated secondary antibody (Sigma) and the ECL system (GE Healthcare).

### Lambda phosphatase treatment.

Protein dephosphorylation assays were performed essentially as previously described in reference 34 with slight modifications. Total protein extracts were prepared from 1.5 × 10^8^ cells. Twenty-five-milliliter cultures were mixed with 2.5 ml of 100% (wt/vol) trichloroacetic acid and incubated on ice for 30 min. Cells were centrifuged, and the pellets were washed once with 10 ml of ice-cold acetone and twice with 500 μl of beating buffer (8 M urea, 50 mM ammonium bicarbonate) containing a protease inhibitor cocktail (Sigma-Aldrich). Cells were resuspended in 200 μl of beating buffer plus protease inhibitors and disrupted with 0.5-mm glass beads in a FastPrep cell disruptor for three cycles of 35 s at 5.5 m/s, 4°C. For assays, 150 μg of protein was treated with 400 U of λ-protein phosphatase (New England Biolabs) in the presence/absence of specific phosphatase inhibitor (5 mM sodium orthovanadate) for 50 min at 30°C. The reaction volume was adjusted with water to dilute the urea-containing buffer at a ratio of 1:10. Protein electrophoresis was performed on 10% SDS-PAGE gels, and the Rnc1-HA fusion was detected as indicated.

### *In vitro* kinase assay.

GST-Wis1DD (constitutively active MAPKK), GST-Sty1 (T97A) (analog-sensitive MAPK), GST-Rnc1, GST-Rnc1(T50A), and GST-Rnc1(S/T6A) fusions were purified from Escherichia coli with glutathione-Sepharose 4B beads (GE Healthcare, USA). After washing extensively, GST-Wis1DD, GST-Sty1 (T97A), and GST-Rnc1 substrates were incubated in 20 mM Tris (pH 8), 10 mM MgCl_2_, and 20 μM ATPγS at 30°C for 45 min in the presence/absence of 20 μM kinase-specific inhibitor BrB-PP1 (Abcam). The kinase reaction was stopped by adding 20 mM EDTA, and the reaction mixture was alkylated after incubatiion at room temperature with 2.5 mM *p*-nitrobenzyl mesylate for 1 h. Rnc1 phosphorylation was detected using an antibody to thiophosphate ester (Abcam; 92570). GST fusions were detected with anti-GST antibody (GE Healthcare, USA).

### Quantification of Western blot experiments and reproducibility of results.

Densitometric quantification of Western blot signals as 16-bit .jpg digital images of blots was performed using ImageJ (35). The desired bands plus background were drawn as rectangles, and a profile plot was obtained for each band (peaks). To minimize the background noise in the bands, each peak floating above the baseline of the corresponding peak was manually closed off using the straight-line tool. Finally, measurement of the closed peaks was performed with the wand tool. Relative units for Sty1 and Pmk1 activation were estimated by determining the signal ratio of the anti-phospho-P38 (activated Sty1) and anti-phospho-P44/42 (activated Pmk1) blots with respect to the anti-HA blot (total Sty1 or Pmk1) at each time point. Relative units for total Mcs4, Wak1, Wis1, Atf1, Pyp1, Pyp2, and Ptc1 levels were estimated by determining the signal ratio of the corresponding anti-HA (total Rnc1), anti-GFP (total Mcs4), anti-Atf1, or anti-c-*myc* (total Wak1, Wis1, Pyp1, Pyp2, and Ptc1) blots with respect to the anti-Cdc2 blot (internal control) at each time point. Unless otherwise stated, results shown correspond to experiments performed as biological triplicates. Mean relative units ± SD and/or representative results are shown. *P* values were analyzed by unpaired Student’s *t* test.

### Plate assays of stress sensitivity for growth and cell recovery after acute thermal stress.

In the growth sensitivity assay, S. pombe wild-type and mutant strains were grown in YES liquid medium to an OD_600_ of 0.5, and appropriate decimal dilutions were spotted per triplicate on YES solid medium or in the same medium supplemented with various concentrations of potassium chloride, sodium chloride, hydrogen peroxide, or caffeine (all from Sigma-Aldrich). Plates were incubated at either 30 or 37°C for 3 days and then photographed. In the growth recovery assay after acute stress, decimal dilutions of strains were spotted per triplicate on YES solid medium, and the plates were allowed to dry at room temperature for 10 min and incubated in an oven at 55°C. The plates were removed from the oven at timed intervals (0 to 120 min), incubated for 3 days at 30°C, and then photographed. All the assays were repeated at least three times with similar results. Representative experiments are shown in the corresponding figures.

### Microscopy analysis.

Fluorescence images were obtained with a Leica DM4000B microscope equipped with a Leica DC400F camera and processed using IM500 Image Manager software. Calcofluor white was employed for cell wall/septum as described previously (13). To determine cell length at division, the yeast strains were grown in YES medium to an *A*_600_ of 0.5 and stained with calcofluor white. A minimum of 200 septated cells were scored for each strain. Three biological replicates were scored for each strain genotype.
