# Supplementary material for: RNA-Binding Protein Rnc1 Regulates Cell Length at Division and Acute Stress Response in Fission Yeast through Negative Feedback Modulation of the Stress-Activated Mitogen-Activated Protein Kinase Pathway
Source: mBio. 2020 Jan 7;11(1):e02815-19. doi: 10.1128/mBio.02815-19 (PMC6946801; doi:10.1128/mBio.02815-19)
Supplement: TABLE S1 [file mBio.02815-19-st001.docx]

**Table S1.** *S. pombe* strains used in this study.

| **Strain** | **Genotype** | **Source/Reference** |
| --- | --- | --- |
| MI200 | h^+^ *pmk1-HA6H::ura4^+^* *ade6-M216 leu1-32 ura4D-18* | Madrid *et al.,* 2006 |
| E086 | h^+^ *rnc1::kanR* *pmk1-HA6H::ura4^+^* *ade6-M216 leu1-32 ura4D-18* | This work |
| MI100 | h^+^ *pmk1::kanR* *sty1-HA6H::ura4^+^* *ade6-M216 leu1-32 ura4D-18* | Madrid *et al.,* 2007 |
| E118 | h^+^ *pmk1::natR* *rnc1::kanR sty1-HA6H::ura4^+^* *ade6-M216 leu1-32 ura4D-18* | This work |
| MI212 | h^+^ *pmp1::kanR* *pmk1-HA6H::ura4^+^* *ade6-M216 leu1-32 ura4D-18* | Madrid *et al.,* 2007 |
| 2119 | h^-^ *his7-336* *wis1DD-12myc::ura4^+^* *ade6-M216 leu1-32 ura4D-18* | M.A. Rodriguez-Gabriel |
| E137 | h^+^ *wis1DD-12myc::ura4^+^* *rnc1::kanR* *ade6-M216 leu1-32 ura4D-18* | This work |
| MM516 | h^+^ *sty1::ura4^+^* *pmk1-HA6H::ura4^+^* *ade6-M216 leu1-32 ura4D-18* | Madrid *et al.,* 2007 |
| FPR086 | h*^-^ sty1::ura4^+^ rnc1::kanR pmk1-HA6H::ura4^+^* *ade6-M216 leu1-32 ura4D-18* | This work |
| JM1521 | h^+^ *sty1-HA6H::ura4^+^* *ade6-M216 leu1-32 ura4D-18* | J.B.A. Millar |
| E088 | h^-^ *rnc1::kanR* *sty1-HA6H::ura4^+^* *ade6-M216 leu1-32 ura4D-18* | This work |
| PPG148 | h^-^ *cdc25-22 ura4D-18* | Madrid *et al.,* 2006 |
| FPR176 | h^+^ *cdc25-22 rnc1::kanR* *ade6-M216 leu1-32 ura4D-18* | This work |
| FPR483 | h^+^ *his7-336* *wak1-9myc::ura4^+^* *ade6-M216 leu1-32 ura4D-18* | This work |
| FPR484 | h^?^ *his7-336* *rnc1::kanR wak1-9myc::ura4^+^* *ade6-M216 leu1-32 ura4D-18* | This work |
| E312 | h^+^ *mcs4-GFP::kanR*  *ade6-M216 leu1-32 ura4D-18* | This work |
| FPR074 | h^+^ *mcs4-GFP::kanR rnc1::natR* *ade6-M216 leu1-32 ura4D-18* | This work |
| KS2079 | h^-^ *wis1-12myc::ura4^+^* *ade6-M216 leu1-32 ura4D-18* | M.A. Rodriguez-Gabriel |
| E010 | h^+^ *wis1-12myc::ura4^+^ rnc1::kanR ade6-M216 leu1-32 ura4D-18* | This work |
| MM1 | h^+^ *ade6-M216 leu1-32 ura4D-18* | Madrid *et al.,* 2006 |
| FPR046 | h*^-^ rnc1::kanR ade6-M216 leu1-32 ura4D-18* | This work |
| MI701 | h^+^ *pyp1-13myc::kanR* *ade6-M216 leu1-32 ura4D-18* | Madrid *et al.,* 2007 |
| E090 | h^+^ *pyp1-13myc::kanR rnc1::hygR* *ade6-M216 leu1-32 ura4D-18* | This work |
| MI702 | h^+^ *pyp2-13myc::ura4^+^* *ade6-M216 leu1-32 ura4D-18* | Madrid *et al.,* 2007 |
| E092 | h^-^ *pyp2-13myc::ura4^+^ rnc1::kanR* *ade6-M216 leu1-32 ura4D-18* | This work |
| MI703 | h^+^ *ptc1-13myc::kanR* *ade6-M216 leu1-32 ura4D-18* | Madrid *et al.,* 2007 |
| FPR078 | h^+^ *ptc1-13myc::kanR rnc1::natR* *ade6-M216 leu1-32 ura4D-18* | This work |
| MI1001 | h^+^ *his7-336* *pyp1::kanR* *sty1-HA6H::ura4^+^* *ade6-M216 leu1-32 ura4D-18* | Madrid *et al.,* 2007 |
| FPR296 | h^?^ *rnc1::kanR pyp1::kanR* *sty1-HA6H::ura4^+^* *ade6-M216 leu1-32 ura4D-18* | This work |
| MI704 | h^+^ *pyp2-13myc::ura4^+^ pyp1::kanR* *ade6-M216 leu1-32 ura4D-18* | Madrid *et al.,* 2007 |
| FPR297 | h^?^ *rnc1::kanR pyp1::kanR* *pyp2-13myc::ura4^+^* *ade6-M216 leu1-32 ura4D-18* | This work |
| FPR101 | h^+^ *rnc1-3HA::kanR*  *ade6-M216 leu1-32 ura4D-18* | This work |
| FPR177 | h*^-^ sty1::ura4^+^ sty1-GFP::leu1^+^* *ade6-M216 leu1-32 ura4D-18* | This work |
| FPR183 | h^?^ *sty1::ura4^+^ rnc1-3HA::kanR sty1-GFP::leu1^+^* *ade6-M216 leu1-32 ura4D-18* | This work |
| FPR322 | *h^+^ rnc1(T45AT50AT171AT177AS278AS286A)-3HA::kanR*  *ade6-M216 leu1-32 ura4D-18* | This work |
| FPR235 | h*^+^ cdc25-22 rnc1-3HA::kanR ade6-M216 leu1-32 ura4D-18* | This work |
| FPR244 | h*^?^ cdc25-22 rnc1-3HA::kanR pmk1::natR ade6-M216 leu1-32 ura4D-18* | This work |
| FPR102 | h^?^ *rnc1-3HA::kanR pmk1::natR*  *ade6-M216 leu1-32 ura4D-18* | This work |
| FPR110 | h^?^ *rnc1-3HA::kanR sty1::ura4^+^*  *ade6-M216 leu1-32 ura4D-18* | This work |
| FPR112 | h^?^ *rnc1-3HA::kanR pmk1::kanR sty1::ura4^+^*  *ade6-M216 leu1-32 ura4D-18* | This work |
| FPR321 | h^+^ *rnc1(K111DA112DR196DN197DR338DG339D)-3HA::kanR*  *ade6-M216 leu1-32 ura4D-18* | This work |
| FPR197 | *h^+^ cdc10-129 rnc1-3HA::kanR ade6-M216 leu1-32 ura4D-18* | This work |
| FPR396 | *h^?^ nda3-km311 rnc1-3HA::kanR ade6-M216 leu1-32 ura4D-18* | This work |
| FPR124 | h^?^ *rnc1-3HA::kanR sty1-HA6H::ura4^+^*  *ade6-M216 leu1-32 ura4D-18* | This work |
| FPR422 | h^?^ *rnc1(K111DA112DR196DN197DR338DG339D)-3HA::kanR sty1-HA6H::ura4^+^*  *ade6-M216 leu1-32 ura4D-18* | This work |
| FPR424 | h^?^ *rnc1(T45AT50AT171AT177AS278AS286A)-3HA::kanR sty1-HA6H::ura4^+^*  *ade6-M216 leu1-32 ura4D-18* | This work |
| FPR122 | h^?^ *rnc1-3HA::kanR pmk1-HA6H::ura4^+^*  *ade6-M216 leu1-32 ura4D-18* | This work |
| FPR423 | h^?^ *rnc1(K111DA112DR196DN197DR338DG339D)-3HA::kanR pmk1-HA6H::ura4^+^*  *ade6-M216 leu1-32 ura4D-18* | This work |
| FPR425 | h^?^ *rnc1(T45AT50AT171AT177AS278AS286A)-3HA::kanR pmk1-HA6H::ura4^+^*  *ade6-M216 leu1-32 ura4D-18* | This work |
| FPR486 | h^?^ *rnc13HA::kanR wak1-9myc::ura4^+^*  *ade6-M216 leu1-32 ura4D-18* | This work |
| FPR489 | h^?^ *rnc1(K111DA112DR196DN197DR338DG339D)-3HA::kanR wak1-9myc::ura4^+^*  *ade6-M216 leu1-32 ura4D-18* | This work |
| FPR492 | h^?^ *rnc1(T45AT50AT171AT177AS278AS286A)-3HA::kanR wak1-9myc::ura4^+^*  *ade6-M216 leu1-32 ura4D-18* | This work |
| FPR416 | h^?^ *rnc13HA::kanR wis1-12myc::ura4^+^*  *ade6-M216 leu1-32 ura4D-18* | This work |
| FPR417 | h^?^ *rnc1(K111DA112DR196DN197DR338DG339D)-3HA::kanR wis1-12myc::ura4^+^*  *ade6-M216 leu1-32 ura4D-18* | This work |
| FPR418 | h^?^ *rnc1(T45AT50AT171AT177AS278AS286A)-3HA::kanR wis1-12myc::ura4^+^*  *ade6-M216 leu1-32 ura4D-18* | This work |
| FPR426 | h^?^ *rnc1-3HA::kanR pyp1-13myc::kanR*  *ade6-M216 leu1-32 ura4D-18* | This work |
| FPR427 | h^?^ *rnc1(K111DA112DR196DN197DR338DG339D)-3HA::kanR pyp1-13myc::kanR*  *ade6-M216 leu1-32 ura4D-18* | This work |
| FPR428 | h^?^ *rnc1(T45AT50AT171AT177AS278AS286A)-3HA::kanR pyp1-13myc::kanR* *ade6-M216 leu1-32 ura4D-18* | This work |
| FPR429 | h^?^ *rnc1-3HA::kanR pyp2-13myc::kanR*  *ade6-M216 leu1-32 ura4D-18* | This work |
| FPR430 | h^?^ *rnc1(K111DA112DR196DN197DR338DG339D)-3HA::kanR pyp2-13myc::kanR*  *ade6-M216 leu1-32 ura4D-18* | This work |
| FPR431 | h^?^ *rnc1(T45AT50AT171AT177AS278AS286A)-3HA::kanR pyp2-13myc::kanR* *ade6-M216 leu1-32 ura4D-18* | This work |
