# Supplementary material for: RNA-Binding Protein Rnc1 Regulates Cell Length at Division and Acute Stress Response in Fission Yeast through Negative Feedback Modulation of the Stress-Activated Mitogen-Activated Protein Kinase Pathway
Source: mBio. 2020 Jan 7;11(1):e02815-19. doi: 10.1128/mBio.02815-19 (PMC6946801; doi:10.1128/mBio.02815-19)
Supplement: TABLE S2 [file mBio.02815-19-st002.docx]

**Table S2.** Oligonucleotides and DNA fragments used in this study.

| **OLIGONUCLEOTIDE** | **SEQUENCE 5’-3’** | **Use** |
| --- | --- | --- |
| Rnc1D-FWD | AATTTAGAAAAGGTTCTACTCTCCTCCTAGAAACGCATATGTGTCCTATTCAATTAACCAATACTATTCCAGTGACATCTCGGATCCCCGGGTTAATTAA | *rnc1^+^* deletion |
| Rnc1D-REV | ACACAAGTCCAAAAAAATCTAGCAAACAAGAGAGAATACCTTGGATGCAAAACCAAAGTACGAAGGCAAGTACTAAGAGTGAATTCGAGCTCGTTTAAAC | *rnc1^+^* deletion |
| Rnc1D-COMP FWD | ACTTCCTATCAGTAAATTGTCGAC | Confirmation of *rnc1^+^* deletion |
| Rnc1-CT-FUSION-FWD | ACACACGAGGAAAATGAGAAAGCCCTTTTCTTACTCTACCAGCAATTAGAAATGGAAAAAGATCGTCGTTCTCATCGGATCCCCGGGTTAATTAA | *rnc1^+^* C-terminal tagging |
| Rnc1-CT-FUSION-REV | TGTGACACCACTAGTTAAACTAAACCAAATTTTTACAAAACATTGATGAACGGGAAAGGGGAAAAGCAAAAGGATAAACTGAATTCGAGCTCGTTTAAAC | *rnc1^+^* C-terminal tagging |
| Rnc1-CT-COMP-F | GTTGGTTGTATAATAGGTCGTGGAG | Confirmation of *rnc1^+^* tagging |
| KAN-COMP-R | GATGTGAGAACTGTATCCTAGCAAG | Common oligonucleotide for confirmation of gene tagging |
| Rnc1-Casette FWD | TTCACTTCCAAAGATATAACGACTGAGGCG | C-terminal tagged *rnc1^+^*cassette amplification, cloning and sequencing |
| Rnc1-Casette REV | CCAATATTCATGCAACAACGTATAGAGCTG | C-terminal tagged *rnc1^+^*cassette amplification and cloning |
| Rnc1-Seq1 FWD | CAAGTCTCCCCTCCAGCAGCTCCC | *rnc1^+^* sequencing |
| Rnc1-Seq2 FWD | TTCTATGAACTGCGGTGTTACATAG | *rnc1^+^* sequencing |
| Rnc1-T50A-FWD | CATTGCTAAAGTTTCCATACCTACTCCAAAGCCCTCTGCACCTCTATCGACTCTTACTAACGGTTCTACTATTCAACAGT | Rnc1 threonine-50 replaced by alanine (site-directed mutagenesis) |
| Rnc1-T50A-REV | ACTGTTGAATAGTAGAACCGTTAGTAAGAGTCGATAGAGGTGCAGAGGGCTTTGGAGTAGGTATGGAAACTTTAGCAATG | Rnc1 threonine-50 replaced by alanine (site-directed mutagenesis) |
| GSTRnc1-FWD-BamHI | TTAATGGATCCATGGCTTACAATCACTTCAGCATTC | Cloning of *rnc1^+^* ORF into pGEX-KG |
| GSTRnc1-REV-XbaI | TTAATTCTAGATCAATGAGAACGACGATCTTTTTCC | Cloning of *rnc1^+^* ORF into pGEX-KG |
| GSTSty1-FWD-SmaI | TTAATCCCGGGAATGGCAGAATTTATTCGTACACAAAT | Cloning of *sty1^+^* ORF into pGEX-KG |
| GSTSty1-REV-XbaI | TTAATTCTAGAATGGATTGCAGTTCATTATCCATGTTG | Cloning of *sty1^+^* ORF into pGEX-KG |
| GST-REV-BamHI | TTAATTCTAGATCAGTCACGATGAATAAGCTTGAG | Cloning of GST and GST tagged *rnc1^+^* into pREP3X |
| GST-FWD-XhoI | TTAATCTCGAGATGTCCCCTATACTAGGTTATTGGA | Cloning of GST and GST tagged *rnc1^+^* into pREP3X |
| GST-Rnc1-REV-SmaI | TTAATCCCGGGTCAATGAGAACGACGATCTTTTTCC | Cloning of GST and GST tagged *rnc1^+^* into pREP3X |
| Leu1-FWD | CTTCCCTTCTCCTTCGTTATGG | q-PCR |
| Leu2-REV | CCTCCCAAATCGCGAGTATAAA | q-PCR |
| Mcs4-FWD | TTTCCTCGGAGGTTGCTAAAG | q-PCR |
| Mcs4-REV | CATCGTGGGAAGTTGGATGT | q-PCR |
| Wis4-FWD | AAACCCAGAAGCACTGAAGG | q-PCR |
| Wis4-REV | CTATCGGATGAACGGGACATAAA | q-PCR |
| Win1-FWD | TGATACGACAAAGGAGAACAGG | q-PCR |
| Win1-REV | CCGAAAGAACCGCTACCTATAA | q-PCR |
| Wis1-FWD | ATCTGGCTCTTCGTTTCGTATT | q-PCR |
| Wis1-REV | GTCGGTTGATGCAATGCTTTAT | q-PCR |
| Sty1-FWD | ATGACGGGCTATGTTTCTACTC | q-PCR |
| Sty1-REV | ATACAACCCGCACTCCAAATA | q-PCR |
| Atf1-FWD | TCACCTGGTACTGCCAATTTAT | q-PCR |
| Atf1-REV | CCATTTACAACAGGCGGTTTAC | q-PCR |
| Pyp1-FWD | GAAGGCTCCGATTACTTCTCTC | q-PCR |
| Pyp1-REV | TGTTGTCCTTGTTCTCAGGTAG | q-PCR |
| Pyp2-FWD | CTACGATCGGTGCCTTCTTATC | q-PCR |
| Pyp2-REV | TGACGACGTTGCTGGATTTA | q-PCR |
| Ptc1-FWD | CGCTGCAGTTGCTTTCTTTAG | q-PCR |
| Ptc1-REV | GCCTTACCATCACGGCATAATA | q-PCR |
| Ptc3-FWD | CGTACTCGCTTGTGATGGTATT | q-PCR |
| Ptc3-REV | AAGAGAGGTTCCAGCAACTATG | q-PCR |
| Pmp1-FWD | GGATAGGTCCCAACATGTCTTT | q-PCR |
| Pmp1-REV | TTCAAGGATGACGATTGATAGGG | q-PCR |

| **gBLOCKS GENE FRAGMENT** | **SEQUENCE 5’-3’** | **Use** |
| --- | --- | --- |
| Rnc1 (T45A, T50A, T171A, T177A, S278A, S286A) SacI/PacI | TATTA*GAGCTC*TTCACTTCCAAAGATATAACGACTGAGGCGTAAAGCTACGCTACCACTTCCTATCAGTAAATTGTGCGACTTTACTATACGTTCTTCAAACTTCGTTATTTCCCCACCAAAAGACTTACTTGCAGAAAATTTTCTCTTTGTCACTGCTACACCCCGTTTACCACTACTCCGTTCTCCTTGGTCGTTTACTTCATTTTTGTCGAAGTAATCACAGCTATTGATTGCAATTTCAATTTATAAGAAACTGCAATAAGAGCTTAGAAGGAGCCTAATCCGTTTTCCTTTTTTTTTTTAATCTCCGCTAAATCCCTGCAGGTTTGAACTAATCAGTTTTCAGGAAGTAATTTAGAAAAGGTTCTACTCTCCTCCTAGAAACGCATATGTGTCCTATTCAATTAACCAATACTATTCCAGTGACATCTATGGCTTACAATCACTTCAGCATTCCTAAAAACATCGAGGAAAAAGAGAACTCTTTTTTTGACGTAACGTTTCAAGACGAACCCGACGAAACCACTTCTACTGCTACTGGCATTGCTAAAGTTTCCATACCT*GCT*CCAAAGCCCTCT*GCA*CCTCTATCGACTCTTACTAACGGTTCTACTATTCAACAGTCCATGACCAACCAACCCGAACCAACGTCTCAAGTGCCTCCCATCTCTGCCAAGCCACCGATGGATGATGCCACCTATGCTACTCAACAACTTACCTTGAGAGCCTTACTTTCTACTCGTGAAGCTGGTATCATTATTGGTAAAGCTGGAAAAAACGTTGCCGAACTCAGAAGCACTACAAATGTCAAGGCCGGCGTTACCAAGGCTGTTCCTAATGTTCATGATCGTGTTTTAACTATTAGTGGACCACTAGAGAATGTTGTTCGCGCTTATAGATTCATCATCGATATTTTTGCCAAGAACAGTACTAACCCTGATGGT*GCA*CCTTCCGACGCCAAC*GCA*CCTCGCAAACTTCGTCTTTTGATCGCCCATTCTCTGATGGGTAGTATTATTGGCCGCAATGGTTTGCGTATCAAGCTTATTCAGGACAAATGTAGTTGCCGTATGATTGCTTCCAAAGACATGCTTCCACAGTCTACTGAGCGTACAGTTGAAATCCATGGTACAGTCGATAATCTTCATGCTGCCATTTGGGAAATTGGCAAATGCTTAATTGATGACTGGGAGCGTGGCGCCGGTACCGTTTTCTATAATCCCGTTTCTCGTTTGACTCAACCTCTTCCTTCTCTTGCGTCGACTGCA*GCT*CCTCAACAAGTC*GCC*CCTCCAGCAGCTCCCTCCACGACTTCTGGTGAAGCTATCCCCGAAAACTTTGTTTCTTACGGTGCTCAAGTCTTTCCAGCTACCCAAATGCCTTTCTTGCAGCAACCTAAGGTTACCCAAAATATTAGCATTCCCGCAGATATGGTTGGTTGTATAATAGGTCGTGGAGGATCTAAGATTTCGGAAATCCGTCGTACCAGCGGTAGCAAGATTTCCATTGCCAAAGAACCTCATGATGAGACAGGCGAACGTATGTTCACCATTACAGGTACACACGAGGAAAATGAGAAAGCCCTTTTCTTACTCTACCAGCAATTAGAAATGGAAAAAGATCGTCGTTCTCATCGGATCCCCGGG*TTAATTAA*CATCT | Cloning into plasmid pTA-Rnc1:HA to obtain MAPK non-phosphorylatable Rnc1 mutant |
| Rnc1 (K110D, A111D, R196D, N197D, R338D, G339D) SacI/PacI | TATTA*GAGCTC*TTCACTTCCAAAGATATAACGACTGAGGCGTAAAGCTACGCTACCACTTCCTATCAGTAAATTGTGCGACTTTACTATACGTTCTTCAAACTTCGTTATTTCCCCACCAAAAGACTTACTTGCAGAAAATTTTCTCTTTGTCACTGCTACACCCCGTTTACCACTACTCCGTTCTCCTTGGTCGTTTACTTCATTTTTGTCGAAGTAATCACAGCTATTGATTGCAATTTCAATTTATAAGAAACTGCAATAAGAGCTTAGAAGGAGCCTAATCCGTTTTCCTTTTTTTTTTTAATCTCCGCTAAATCCCTGCAGGTTTGAACTAATCAGTTTTCAGGAAGTAATTTAGAAAAGGTTCTACTCTCCTCCTAGAAACGCATATGTGTCCTATTCAATTAACCAATACTATTCCAGTGACATCTATGGCTTACAATCACTTCAGCATTCCTAAAAACATCGAGGAAAAAGAGAACTCTTTTTTTGACGTAACGTTTCAAGACGAACCCGACGAAACCACTTCTACTGCTACTGGCATTGCTAAAGTTTCCATACCTACTCCAAAGCCCTCTACACCTCTATCGACTCTTACTAACGGTTCTACTATTCAACAGTCCATGACCAACCAACCCGAACCAACGTCTCAAGTGCCTCCCATCTCTGCCAAGCCACCGATG*GATGAT*GCCACCTATGCTACTCAACAACTTACCTTGAGAGCCTTACTTTCTACTCGTGAAGCTGGTATCATTATTGGT*GATGAT*GGAAAAAACGTTGCCGAACTCAGAAGCACTACAAATGTCAAGGCCGGCGTTACCAAGGCTGTTCCTAATGTTCATGATCGTGTTTTAACTATTAGTGGACCACTAGAGAATGTTGTTCGCGCTTATAGATTCATCATCGATATTTTTGCCAAGAACAGTACTAACCCTGATGGTACACCTTCCGACGCCAACACACCTCGCAAACTTCGTCTTTTGATCGCCCATTCTCTGATGGGTAGTATTATTGGCGACGATGGTTTGCGTATCAAGCTTATTCAGGACAAATGTAGTTGCCGTATGATTGCTTCCAAAGACATGCTTCCACAGTCTACTGAGCGTACAGTTGAAATCCATGGTACAGTCGATAATCTTCATGCTGCCATTTGGGAAATTGGCAAATGCTTAATTGATGACTGGGAGCGTGGCGCCGGTACCGTTTTCTATAATCCCGTTTCTCGTTTGACTCAACCTCTTCCTTCTCTTGCGTCGACTGCAACTCCTCAACAAGTCTCCCCTCCAGCAGCTCCCTCCACGACTTCTGGTGAAGCTATCCCCGAAAACTTTGTTTCTTACGGTGCTCAAGTCTTTCCAGCTACCCAAATGCCTTTCTTGCAGCAACCTAAGGTTACCCAAAATATTAGCATTCCCGCAGATATGGTTGGTTGTATAATAGGT*GATGAT*GGATCTAAGATTTCGGAAATCCGTCGTACCAGCGGTAGCAAGATTTCCATTGCCAAAGAACCTCATGATGAGACAGGCGAACGTATGTTCACCATTACAGGTACACACGAGGAAAATGAGAAAGCCCTTTTCTTACTCTACCAGCAATTAGAAATGGAAAAAGATCGTCGTTCTCATCGGATCCCCGGG*TTAATTAA*CATCT | Cloning into plasmid pTA-Rnc1:HA to obtain non-mRNA binding (KH domains) Rnc1 mutant |
